# Supplementary material for: Cultural transmission of attitudes and behaviours from parents, peers and grandparents
Source: PLoS One. 2026 Jan 28;21(1):e0341433. doi: 10.1371/journal.pone.0341433 (PMC12851453; doi:10.1371/journal.pone.0341433)
Supplement: S1 Text — (PDF) [file pone.0341433.s001.pdf]

S1 Text. Network size distribution

Table A. Distribution of participant social networks by size. A network is composed of a student and their family and friends. Data from the 722 networks that only include the student do not inform our results.

Network size	N networks	N individuals
1	722	722
2	364	728
3	345	1035
4	337	1348
5	95	475
6	32	192
7	105	735
8	5	40
9	2	18

Table B. Response rate for the recruitment of family and friends by the student participants.

Agent recruited	Percent of students
1 Friend (on average)	47.8%
Mother	43.8%
Father	36.5%
Maternal Grandmother	13.6%
Maternal Grandfather	8.5%
Paternal Grandmother	6.8%
Paternal Grandfather	5.0%
