# Supplementary material for: Cultural transmission of attitudes and behaviours from parents, peers and grandparents
Source: PLoS One. 2026 Jan 28;21(1):e0341433. doi: 10.1371/journal.pone.0341433 (PMC12851453; doi:10.1371/journal.pone.0341433)
Supplement: S2 Text — (PDF) [file pone.0341433.s002.pdf]

S2 Text. The survey used in this study

1. Do you believe in a god or higher power?
   From 1: No, not at all, to 7: Yes, absolutely

2. What is your religion?
   - No religion (atheist)
   - No religion (agnostic)
   - Catholic
   - Anglican
   - Uniting Church
   - Other Christian
   - Islam
   - Other: (Text box)

3. How often do you take part in religious ceremonies (e.g., attending church/mosque)?
   - Never
   - Occasionally (1-2 times a year)
   - Sometimes (3-11 times a year)
   - Somewhat often (once a month)
   - Often (once a week)
   - Very often (several times a week)
   - Every day

4. Do you view yourself as more left-wing (progressive) or more right-wing (conservative?)
   From 1: Left-wing (progressive) to 7: Right-wing (conservative)

5. What is your preferred Australian political party? Name one.
   [Text box]

6. Are you politically active (e.g., do you attend rallies, sign petitions, send letters to your local council o campaign for a political party during election periods)?
   From 1: Not at all politically active, to 7: Highly politically active

7. Are you a fan of a particular spectator team sport? Please choose your favourite.
   - AFL
   - Cricket
   - Basketball
   - Soccer
   - Tennis
   - I am not a fan of any sport
   - Other: [Text box]

8. Are you a fan of a particular sports team or player? If yes, please specify.
   - No

- Yes: [Text box]

9. How often do you exercise (e.g., go to the gym, go for a jog, play a sport, etc)? Tick the option that is most true for you.
    - Almost never
    - Once per month
    - A few times per month
    - Once per week
    - 2-4 times per week
    - 5 or more times per week
    - Every day

10. How important is it to you that you have a healthy lifestyle?
    From 1: Not very important, to 7: Very important

11. What are your main forms of exercise? Tick all that apply.
    - Walking
    - Aerobics, fitness or gym activities
    - Swimming, diving or other water sports
    - Cycling
    - Jogging or running
    - Golf
    - Tennis
    - Netball
    - Basketball
    - Soccer
    - Australian rules football
    - Rugby
    - Dancing
    - Martial arts
    - Other: [Text box]

12. How many hours of sleep do you get per night (on average)?
    - Up to than 4
    - 5-6 hours
    - 7-8 hours
    - 9-10 hours
    - More than 10 hours

13. How many cigarettes do you smoke per day?
    - Zero/I don't smoke
    - 1-5 per day
    - 6-10 per day
    - 11-20 per day
    - More than 20 per day

14. How many alcoholic drinks do you consume per week?

- Zero/I don't drink
- 1-2 drinks per week
- 3-6 drinks per week
- 7-13 drinks per week
- 14 or more per week

15. How would you describe your diet? Check all that apply.
    - No specific diet
    - Pescatarian
    - Vegetarian
    - Vegan
    - Gluten-free
    - Other: [Text box]

16. How often do you eat a serve of fresh fruits or vegetables? Tick the option that is most true for you.
    - Never
    - Less than once a week
    - 1-2 times per week
    - 3-4 times per week
    - 1-2 times per day
    - 3 or more times per day

17. How many hours do you spend watching TV/movies/videos (including online, e.g., on YouTube or Instagram)?
    - Never
    - Up to 2 hours per week
    - 3-6 hours per week
    - 1-2 hours per day
    - 3-4 hours per day
    - 5-7 hours per day
    - 8 or more hours per day

18. How much do you enjoy watching TV/movies/videos (including online)?
    From 1: Not at all, to 7: Very much

19. How many hours do you spend reading for pleasure?
    - Never
    - Up to 2 hours per week
    - 3-6 hours per week
    - 1-2 hours per day
    - 3-4 hours per day
    - 5-7 hours per day
    - 8 or more hours per day

20. How much do you enjoy reading?
    From 1: Not at all, to 7: Very much

21. How many hours do you spend listening to music?
- Never
- 1-2 hours per week
- 3-6 hours per week
- 1-2 hours per day
- 3-4 hours per day
- 5-7 hours per day
- 8 or more hours per day

22. How much do you enjoy listening to music?
From 1: Not at all, to 7: Very much

23. How many close friends do you have?
- None
- 1-2
- 3-4
- 5-6
- 7-8
- 9-10
- 11 or more

24. How many hours do you spend on social media (e.g., Facebook, Twitter, Instagram, etc.)?
- Never
- Up to 2 hours per week
- 3-6 hours per week
- 1-2 hours per day
- 3-4 hours per day
- 5-7 hours per day
- 8 or more hours per day

25. How much of your day do you spend interacting with people face-to-face?
- 0-1 hours per day
- 2-3 hours per day
- 4-5 hours per day
- 6-7 hours per day
- 8-9 hours per day
- 9-10 hours per day
- 11 or more hours per day

26. Are you concerned about the environment?
From 1: Not at all, to 7: Extremely

27. Do you take action to help the environment in any of the following ways? Tick all that apply.
- Donate to environmental groups/causes

- Eat locally produced food
- Limit meat consumption
- Select to offset your carbon footprint when given the option
- Use public transport instead of driving
- Ride or walk instead of driving
- Limit water/electricity use
- Compost vegetable scraps
- Recycle your plastics/glassware/cans etc.
- Use reusable shopping bags
- Switch off household lights/appliances when they're not in use
- Volunteer your time to conservation groups/tree planting charities/etc
