# Supplementary material for: Cultural transmission of attitudes and behaviours from parents, peers and grandparents
Source: PLoS One. 2026 Jan 28;21(1):e0341433. doi: 10.1371/journal.pone.0341433 (PMC12851453; doi:10.1371/journal.pone.0341433)
Supplement: S3 Text — (PDF) [file pone.0341433.s003.pdf]

S3 Text. Cavalli-Sforza et al.'s (1982) survey

Cavalli-Sforza & Feldman's (1982) survey included 38 questions around 6 topics:

Religion

1. Do you attend church?
2. Do you pray?
3. Are you Catholic?
4. Are you Jewish?
5. Are you Protestant?

Politics

6. Are you a registered voter?
7. Are you registered with a political party?
8. Do you identify as a Democrat?
9. Do you identify as a Republican?
10. Do you identify as a Conservative?

Sport

11. Do you practice swimming?
12. Do you practice tennis?
13. Do you jog or run?

Entertainment

14. Do you watch TV?
15. Do you listen to classical music?
16. Do you watch football?
17. Do you watch baseball?
18. Do you like to camp?
19. Do you like to visit art museums?
20. Do you like big parties?
21. Do you attend movies?
22. Do you like adventure movies?
23. Do you like light movies?
24. Do you like serious movies?

Habits

25. Do you have high salt use?
26. Do you routinely check the bill?
27. Are you a morning/evening person?
28. Do you like coffee?
29. Do you like tea?
30. Do you take milk with dinner?
31. Are you a last-minute person?

Beliefs

32. Do you read horoscopes?
33. Do you believe in UFOs?
34. Do you believe in ESP?
35. Do you believe in lucky numbers?
36. Do you believe in ability versus luck?

37. Do you believe in the benefits of jogging for health?
38. Do you believe in benefits of margarine or butter for health?
