# Supplementary material for: Cultural transmission of attitudes and behaviours from parents, peers and grandparents
Source: PLoS One. 2026 Jan 28;21(1):e0341433. doi: 10.1371/journal.pone.0341433 (PMC12851453; doi:10.1371/journal.pone.0341433)
Supplement: S4 Text — (PDF) [file pone.0341433.s004.pdf]

## S4 Text. Numeric recoding answers to text questions

Seven of the 27 questions (Q2, Q5, Q7, Q8, Q11, Q15 and Q27) required a text answer. In questions Q2, Q5, Q7, Q8, and Q15, respondents could select a single option from a list, or type their own answer in a text box. In questions Q11 and Q27, respondents could select as many options as they wished from a list. Text-based questions Q7 and Q8 were not numerically recoded and only analysed as text. Responses for the remaining text-based questions were recoded into numeric ones:

- Q2. Answers to Q2 ("What is your religion?") were recoded into binary values: 0 for people who reported no religion, and 1 for those who reported any religion.
- Q5. Political parties mentioned in response to Q5 ("What is your preferred Australian political party? (name one)") were recoded according to how each party is classified in Wikipedia as a new ordinal variable with 8 levels from 0 = "far left" to 7 "far right", as follows:
    - 0 = "communist", "socialist", "greens"
    - 1 = "labour", "sex"
    - 2 = "reason", "center", "health"
    - 3 = "WA", "democrat"
    - 4 = "coalition", "pap"
    - 5 = "libdem"
    - 6 = "conservative", "christian", "katter", "shooters"
    - 7 = "palmer", "one_nation", "yellow-vest"
    - NA = any other party
- Q15. Free-text Q15 ("How would you describe your diet?") received many varied answers, which were recoded into an ordinal variable with 7 categories, from least to most meat eaten:
    - 0 = "vegan"
    - 1 = "vegetarian"
    - 2 = "flexitarian"
    - 3 = "pescatarian"
    - 4 = "no dietary restriction", "health restrictions"," iron", "optifast", "halal", "kosher", "i-fast", "keto", "paleo", "protein", "healthy"
    - 5 = "no red meat"
    - 6 = "meat"
- Q11 & Q27. Q11 ("What are your main forms of exercise?) and Q27 ("Do you take action to help the environment in any of the following ways?) invited participants to tick all the options in the list that applied to them (12 options were listed for question 11 and 10 options for Q27). We created new numeric variables with the number of options ticked.
