# Supplementary material for: Cultural transmission of attitudes and behaviours from parents, peers and grandparents
Source: PLoS One. 2026 Jan 28;21(1):e0341433. doi: 10.1371/journal.pone.0341433 (PMC12851453; doi:10.1371/journal.pone.0341433)
Supplement: S5 Text — (PDF) [file pone.0341433.s005.pdf]

S5 Text. Descriptive statistics of numeric answers

For each survey question that had numeric answers or answers recoded as numeric (see *S4 Text. Numeric recoding of answers to text questions*), the following pages show: a table of descriptives of the answers given by each agent; a histogram of the responses given by each value; and raw correlation values between the answers of each pair of agents. "Reliability" refers to answers by the student, collected a second time.

Question 1. Do you believe in God? Likert 0 to 6; higher values, stronger belief.

	n	mean	sd	median	min	max	skew	kurtosis	se
Student	1641	2.90	2.19	3	0	6	0.15	-1.45	0.05
Reliability	504	2.74	2.10	3	0	6	0.26	-1.29	0.09
Friend1	1044	2.80	2.15	3	0	6	0.19	-1.37	0.07
Friend2	416	2.79	2.12	3	0	6	0.15	-1.37	0.10
Mother	715	3.95	2.21	5	0	6	-0.65	-1.08	0.08
Father	597	3.29	2.46	4	0	6	-0.22	-1.61	0.10
MatGfather	134	3.37	2.40	4	0	6	-0.29	-1.56	0.21
MatGmother	223	4.43	1.97	5	0	6	-1.07	-0.14	0.13
PatGfather	80	3.26	2.44	4	0	6	-0.24	-1.61	0.27
PatGmother	112	4.17	2.23	5	0	6	-0.84	-0.85	0.21

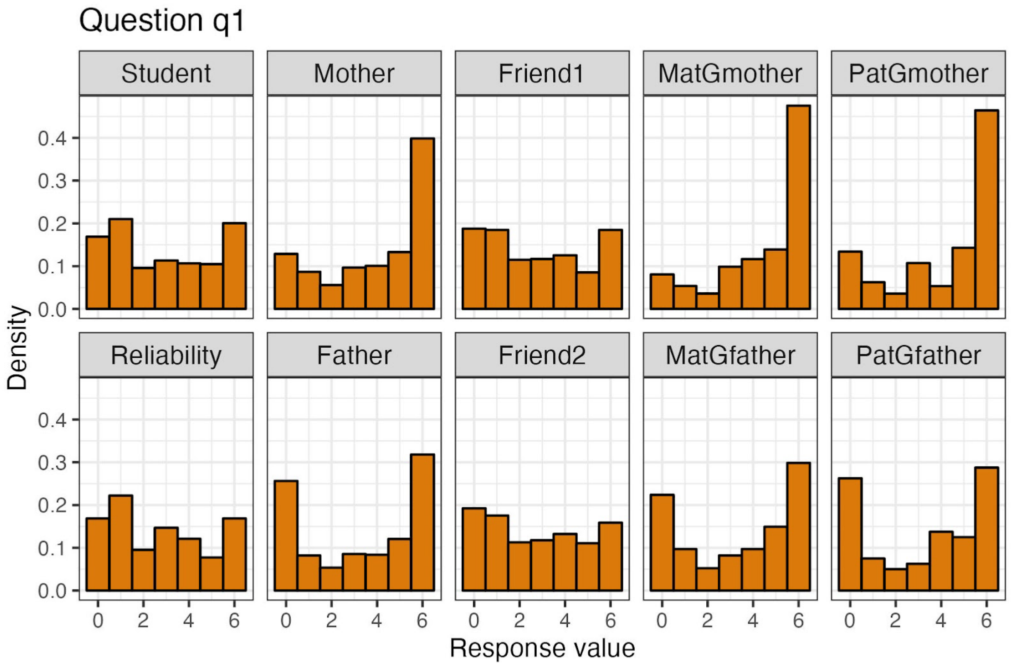

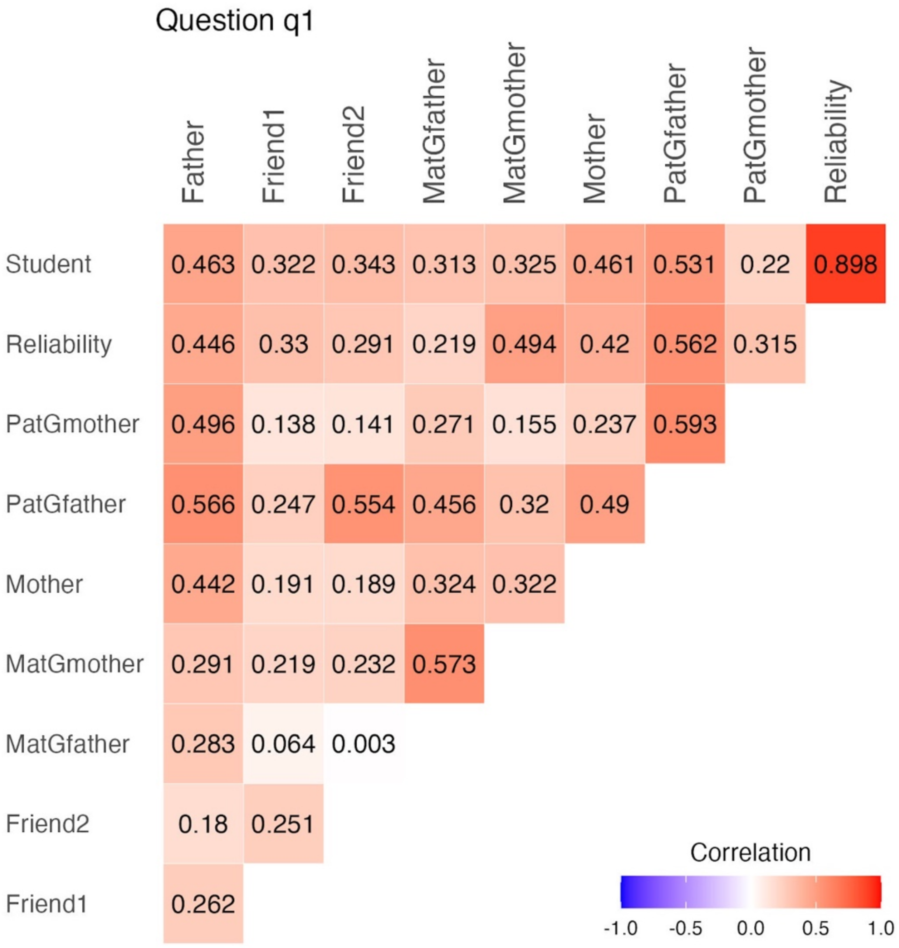

Question 2. What is your religion? Recoded: 0=no religion, agnostic, atheist; 1=any religion

	n	mean	sd	median	min	max	skew	kurtosis	se
Student	1887	0.45	0.50	0	0	1	0.21	-1.96	0.01
Reliability	580	0.44	0.50	0	0	1	0.23	-1.95	0.02
Friend1	1301	0.45	0.50	0	0	1	0.22	-1.95	0.01
Friend2	505	0.44	0.50	0	0	1	0.26	-1.94	0.02
Mother	830	0.70	0.46	1	0	1	-0.87	-1.24	0.02
Father	691	0.63	0.48	1	0	1	-0.54	-1.72	0.02
MatGfather	160	0.69	0.46	1	0	1	-0.80	-1.37	0.04
MatGmother	258	0.80	0.40	1	0	1	-1.48	0.19	0.03
PatGfather	95	0.66	0.48	1	0	1	-0.68	-1.55	0.05
PatGmother	128	0.75	0.43	1	0	1	-1.14	-0.70	0.04

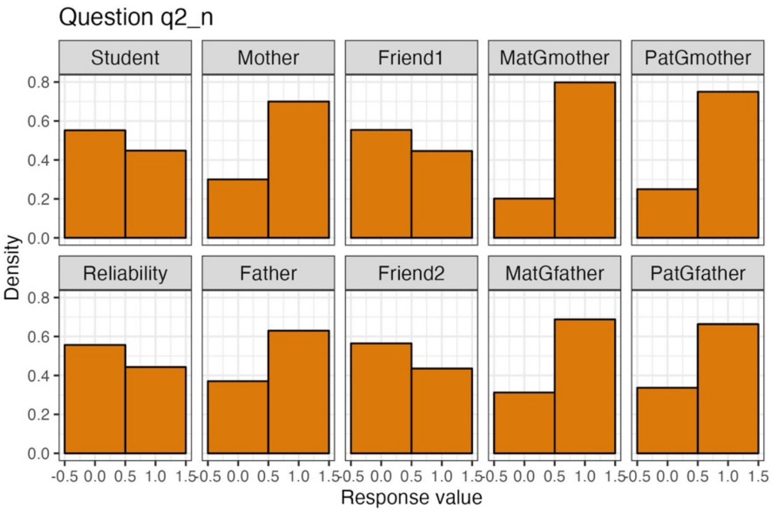
Question q2_n
Student
Mother
Friend1
MatGmother
PatGmother
Reliability
Father
Friend2
MatGfather
PatGfather
Density
0.0
0.2
0.4
0.6
0.8
-0.5 0.0 0.5 1.0 1.5
Response value

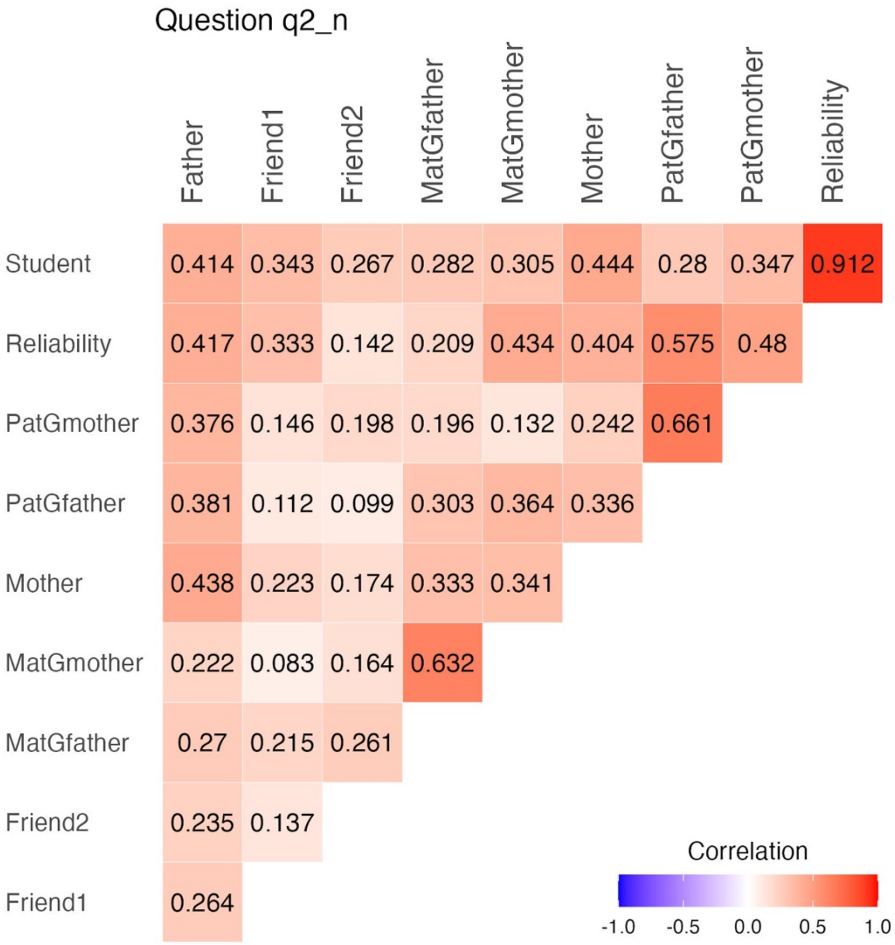
Question q2_n
Father
Friend1
Friend2
MatGfather
MatGmother
Mother
PatGfather
PatGmother
Reliability
Student 0.414 0.343 0.267 0.282 0.305 0.444 0.28 0.347 0.912
Reliability 0.417 0.333 0.142 0.209 0.434 0.404 0.575 0.48
PatGmother 0.376 0.146 0.198 0.196 0.132 0.242 0.661
PatGfather 0.381 0.112 0.099 0.303 0.364 0.336
Mother 0.438 0.223 0.174 0.333 0.341
MatGmother 0.222 0.083 0.164 0.632
MatGfather 0.27 0.215 0.261
Friend2 0.235 0.137
Friend1 0.264
Correlation
-1.0 -0.5 0.0 0.5 1.0

Question 3. How often do you take part in religious ceremonies (e.g., attending church, mosque)?
Likert 0 to 6; higher values, more attendance.

	n	mean	sd	median	min	max	skew	kurtosis	se
Student	1905	1.19	1.46	1	0	6	1.26	0.62	0.03
Reliability	588	1.16	1.41	1	0	6	1.37	1.11	0.06
Friend1	1310	1.15	1.49	1	0	6	1.31	0.68	0.04
Friend2	512	1.07	1.41	1	0	6	1.33	0.75	0.06
Mother	835	1.58	1.70	1	0	6	0.85	-0.50	0.06
Father	695	1.42	1.66	1	0	6	1.05	-0.05	0.06
MatGfather	160	1.56	1.84	1	0	6	0.94	-0.45	0.15
MatGmother	260	2.21	1.94	2	0	6	0.35	-1.29	0.12
PatGfather	96	1.53	1.78	1	0	6	0.92	-0.41	0.18
PatGmother	129	2.25	1.98	2	0	6	0.27	-1.29	0.17

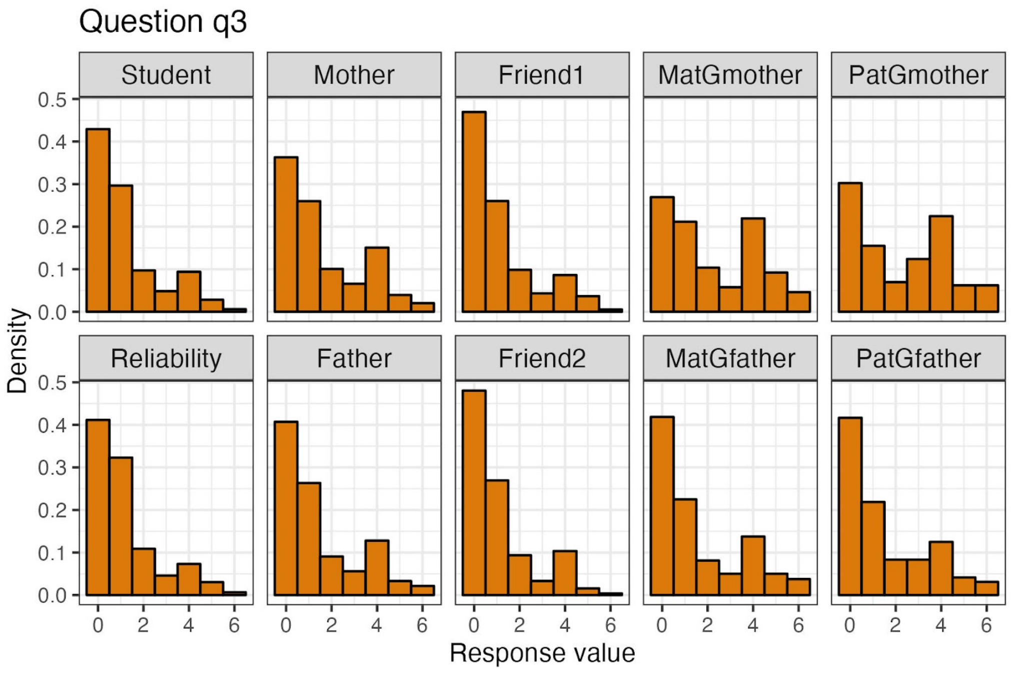

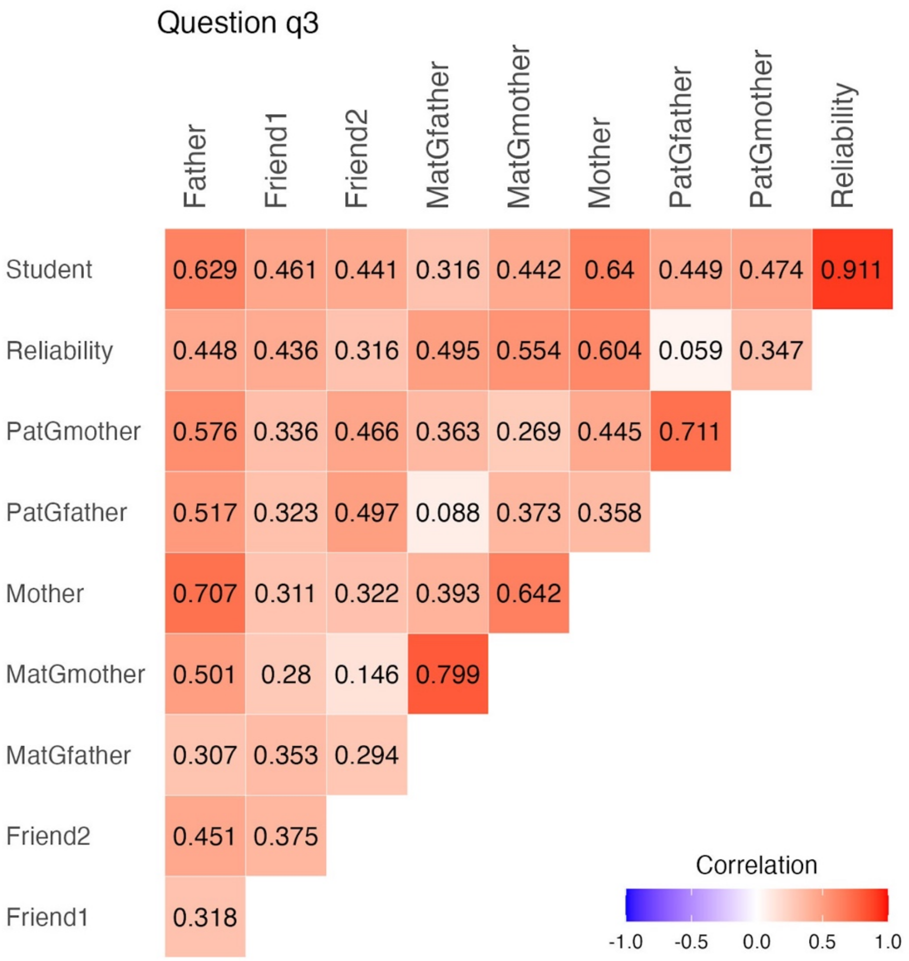

Question 4. Do you see yourself as more left-wing (progressive) or more right-wing (conservative)? Likert 0 to 6; higher values, more conservative.

	n	mean	sd	median	min	max	skew	kurtosis	se
Student	1896	2.00	1.47	2	0	6	0.46	-0.46	0.03
Reliability	581	2.00	1.36	2	0	6	0.34	-0.47	0.06
Friend1	1299	2.12	1.54	2	0	6	0.42	-0.54	0.04
Friend2	508	2.11	1.56	2	0	6	0.45	-0.54	0.07
Mother	828	2.81	1.56	3	0	6	0.07	-0.51	0.05
Father	690	3.10	1.59	3	0	6	-0.12	-0.58	0.06
MatGfather	159	3.53	1.57	4	0	6	-0.46	-0.42	0.12
MatGmother	254	3.40	1.57	3	0	6	-0.37	-0.29	0.10
PatGfather	93	3.58	1.62	4	0	6	-0.52	-0.20	0.17
PatGmother	129	3.60	1.71	4	0	6	-0.47	-0.56	0.15

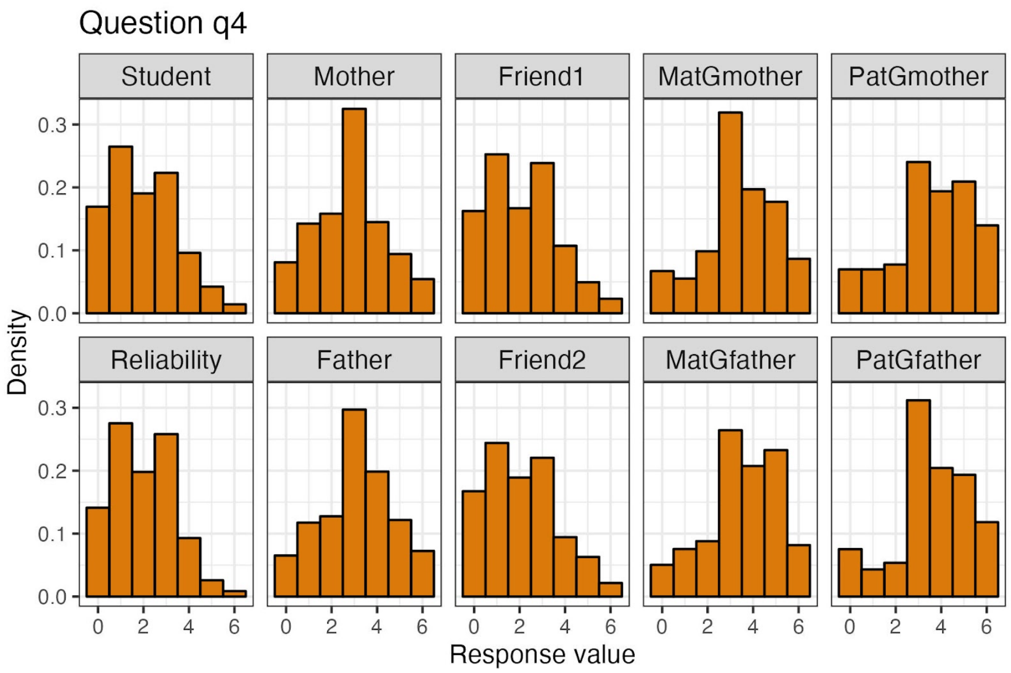

Question q4
Student
Mother
Friend1
MatGmother
PatGmother
Reliability
Father
Friend2
MatGfather
PatGfather
Density
0.0
0.1
0.2
0.3
0
2
4
6
Response value

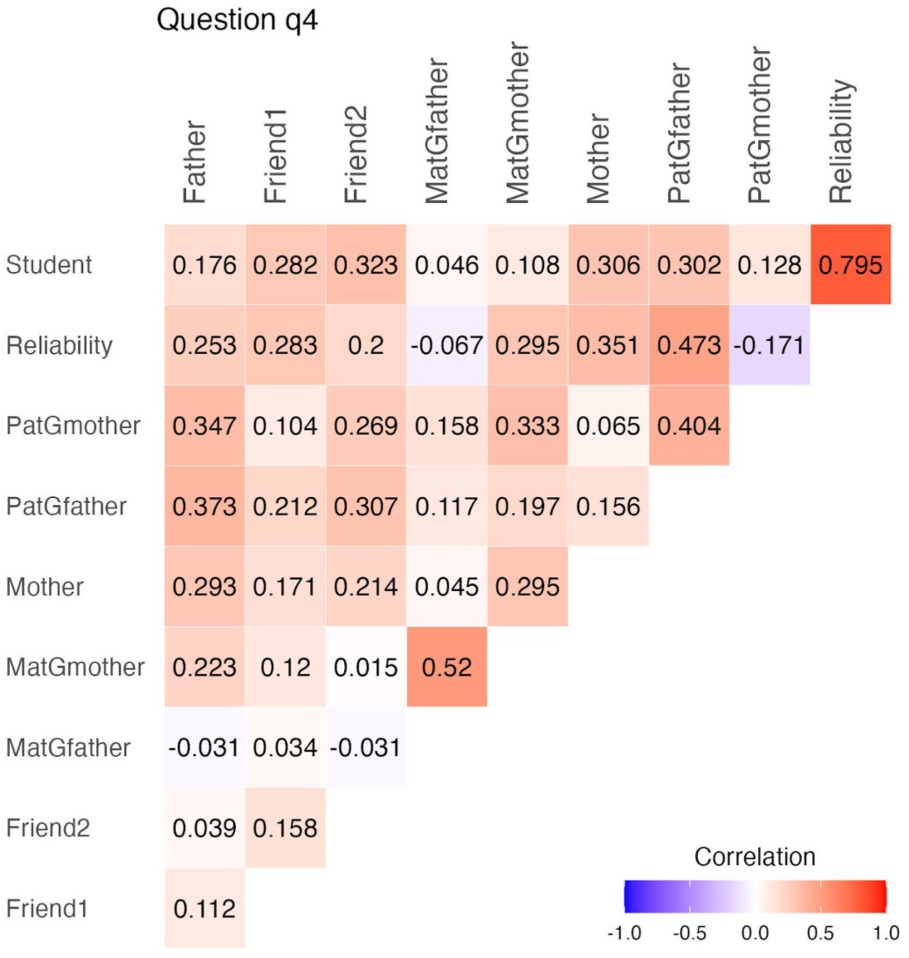

Question q4
Father
Friend1
Friend2
MatGfather
MatGmother
Mother
PatGfather
PatGmother
Reliability
Student 0.176 0.282 0.323 0.046 0.108 0.306 0.302 0.128 0.795
Reliability 0.253 0.283 0.2 -0.067 0.295 0.351 0.473 -0.171
PatGmother 0.347 0.104 0.269 0.158 0.333 0.065 0.404
PatGfather 0.373 0.212 0.307 0.117 0.197 0.156
Mother 0.293 0.171 0.214 0.045 0.295
MatGmother 0.223 0.12 0.015 0.52
MatGfather -0.031 0.034 -0.031
Friend2 0.039 0.158
Friend1 0.112
Correlation
-1.0
-0.5
0.0
0.5
1.0

Question 5. What is your preferred political party? Text-based answer, recoded: 0=extreme left to 7=extreme right, according to Wikipedia's description of political parties (see SM.4).

	n	mean	sd	median	min	max	skew	kurtosis	se
Student	1546	1.55	1.67	1	0	7	0.85	-0.73	0.04
Reliability	488	1.56	1.69	1	0	7	0.76	-0.97	0.08
Friend1	1040	1.66	1.75	1	0	7	0.78	-0.75	0.05
Friend2	394	1.45	1.61	1	0	6	0.96	-0.57	0.08
Mother	656	2.40	1.71	1	0	7	0.04	-1.53	0.07
Father	543	2.76	1.69	4	0	7	-0.16	-1.27	0.07
MatGfather	126	2.88	1.57	4	0	6	-0.39	-1.41	0.14
MatGmother	202	2.85	1.67	4	0	7	-0.24	-1.18	0.12
PatGfather	69	2.59	1.69	4	0	7	0.14	-1.28	0.20
PatGmother	100	2.97	1.75	4	0	7	-0.07	-1.20	0.17

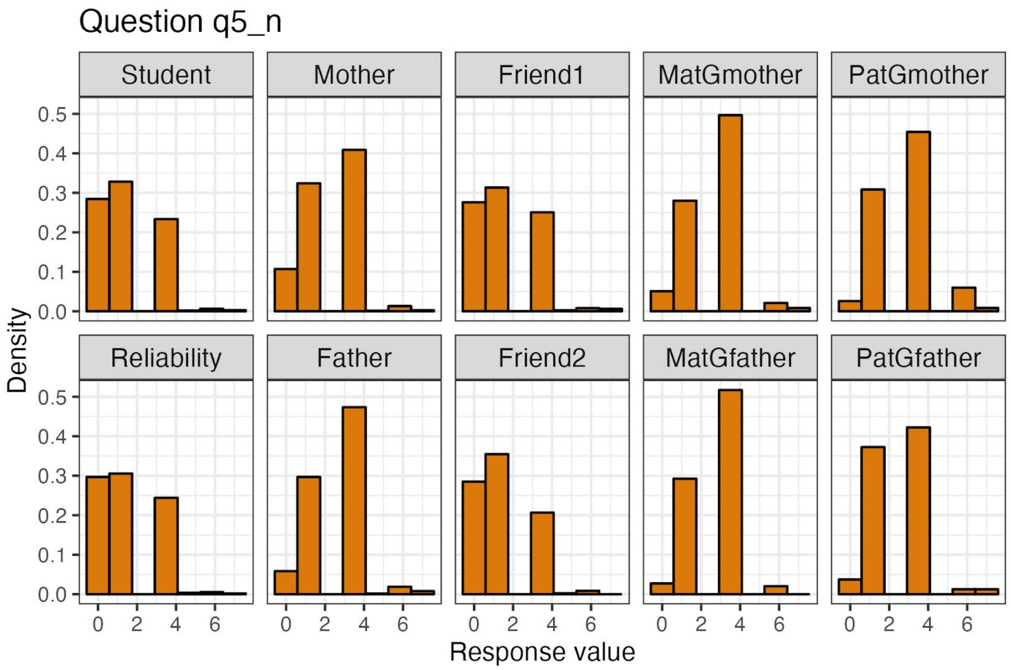

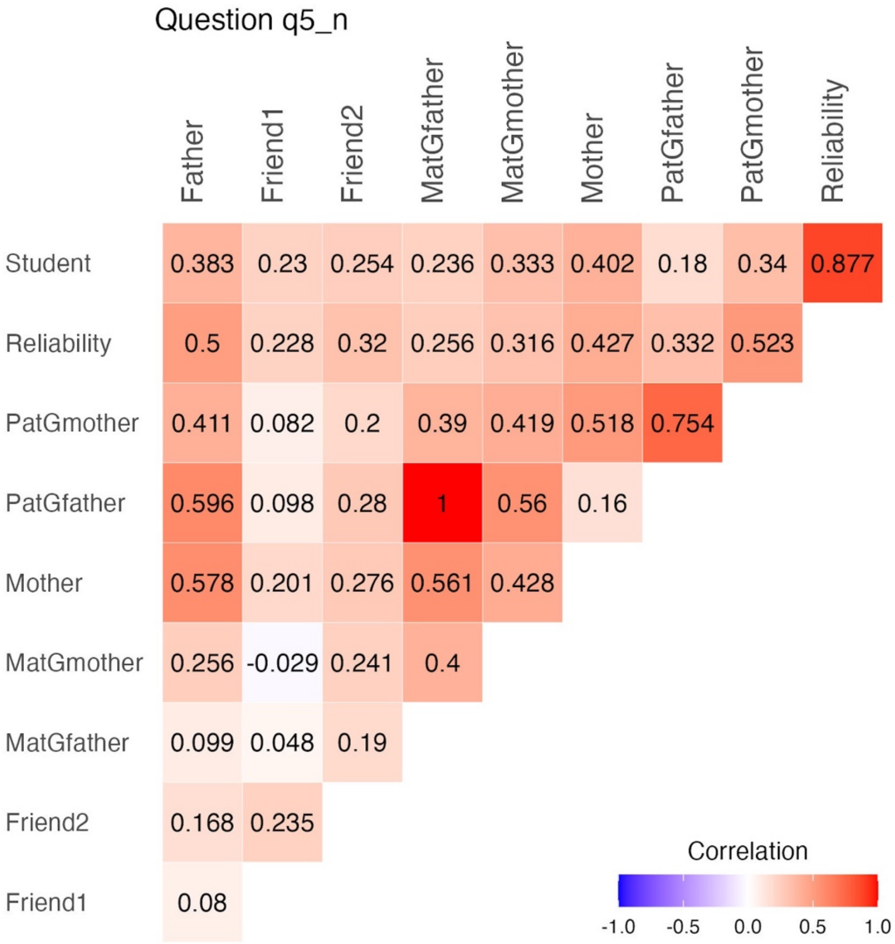

Question 6 - Are you politically active (e.g., attend rallies, sign petitions, send letters to your local council or campaign for a political party during election periods)? Likert 0 to 6; higher values, more activity.

	n	mean	sd	median	min	max	skew	kurtosis	se
Student	1903	1.10	1.45	1	0	6	1.27	0.61	0.03
Reliability	588	1.05	1.39	1	0	6	1.39	1.09	0.06
Friend1	1308	1.04	1.42	0	0	6	1.35	0.91	0.04
Friend2	511	1.08	1.48	0	0	6	1.38	1.07	0.07
Mother	834	0.76	1.31	0	0	6	1.90	2.90	0.05
Father	690	0.87	1.39	0	0	6	1.79	2.50	0.05
MatGfather	159	0.96	1.37	0	0	6	1.56	2.11	0.11
MatGmother	256	0.71	1.19	0	0	6	1.80	2.68	0.07
PatGfather	93	0.95	1.45	0	0	5	1.27	0.30	0.15
PatGmother	130	0.74	1.19	0	0	6	1.85	3.23	0.10

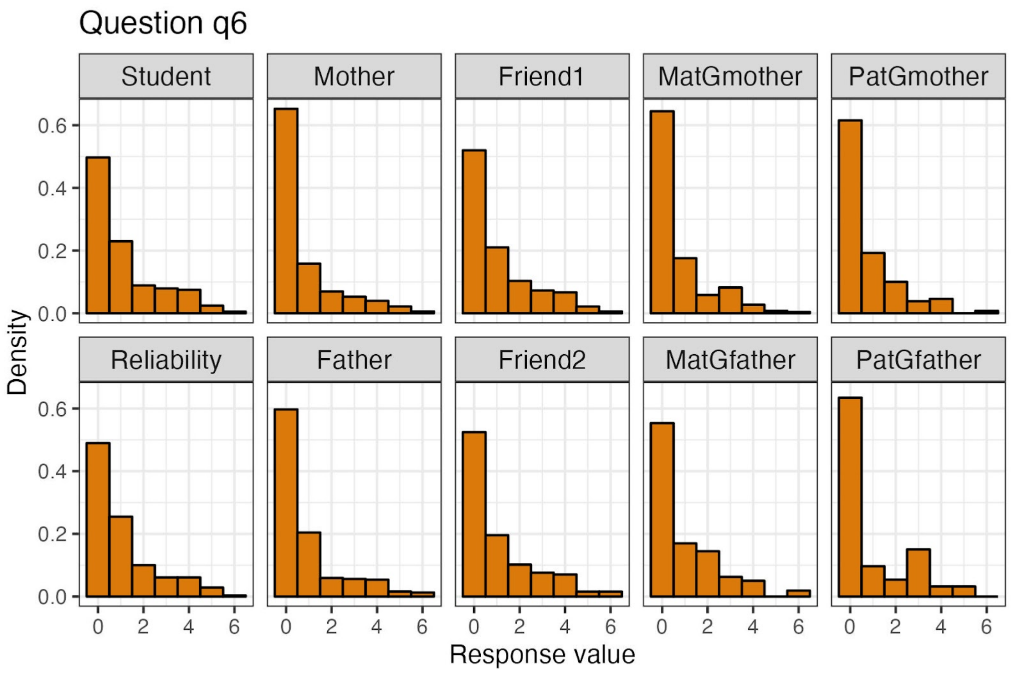
Question q6
Student
Mother
Friend1
MatGmother
PatGmother
Reliability
Father
Friend2
MatGfather
PatGfather
Density
0.0
0.2
0.4
0.6
0
2
4
6
Response value

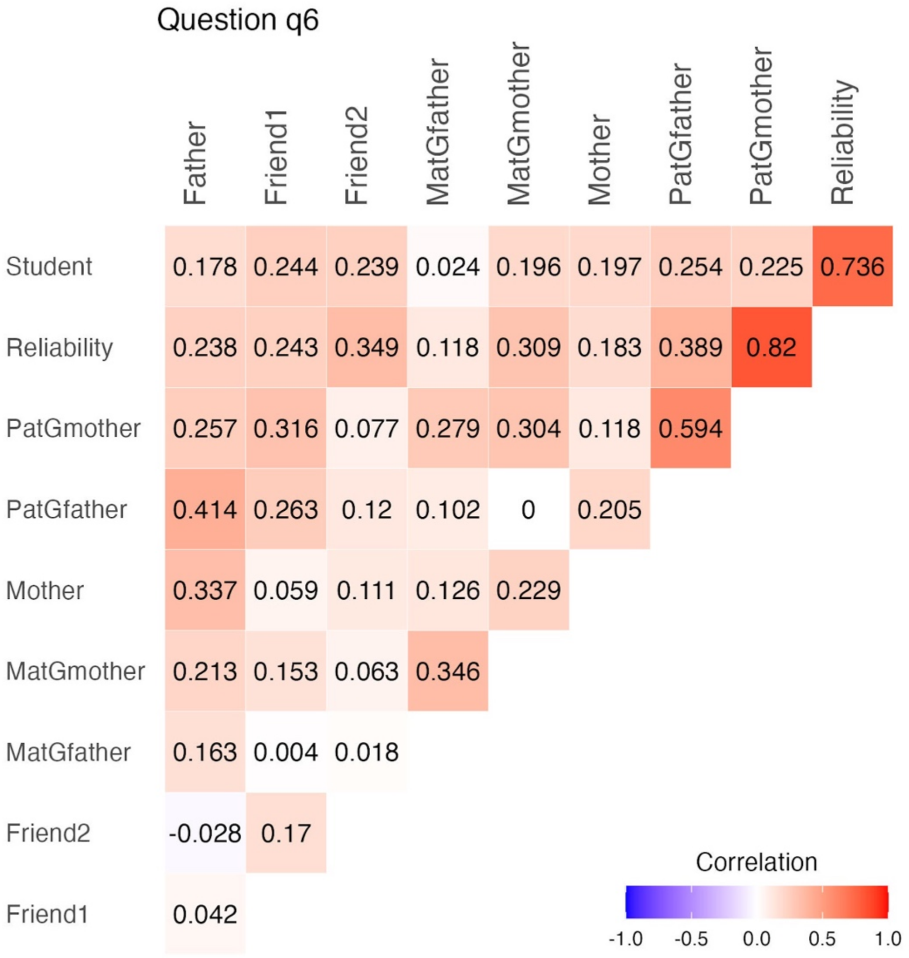
Question q6
Father
Friend1
Friend2
MatGfather
MatGmother
Mother
PatGfather
PatGmother
Reliability
Student 0.178 0.244 0.239 0.024 0.196 0.197 0.254 0.225 0.736
Reliability 0.238 0.243 0.349 0.118 0.309 0.183 0.389 0.82
PatGmother 0.257 0.316 0.077 0.279 0.304 0.118 0.594
PatGfather 0.414 0.263 0.12 0.102 0 0.205
Mother 0.337 0.059 0.111 0.126 0.229
MatGmother 0.213 0.153 0.063 0.346
MatGfather 0.163 0.004 0.018
Friend2 -0.028 0.17
Friend1 0.042
Correlation
-1.0
-0.5
0.0
0.5
1.0

Question 9. How often do you exercise (e.g., go to the gym)? Likert 0 to 6; higher values, more often.

	n	mean	sd	median	min	max	skew	kurtosis	se
Student	1902	3.62	1.44	4	0	6	-0.73	0.31	0.03
Reliability	584	3.65	1.39	4	0	6	-0.74	0.37	0.06
Friend1	1307	3.47	1.58	4	0	6	-0.58	-0.31	0.04
Friend2	512	3.49	1.55	4	0	6	-0.62	-0.16	0.07
Mother	830	3.53	1.76	4	0	6	-0.60	-0.46	0.06
Father	692	3.54	1.78	4	0	6	-0.57	-0.53	0.07
MatGfather	159	3.09	2.15	4	0	6	-0.20	-1.30	0.17
MatGmother	257	2.95	2.13	3	0	6	-0.10	-1.31	0.13
PatGfather	95	2.88	2.22	3	0	6	-0.10	-1.45	0.23
PatGmother	130	3.03	2.08	4	0	6	-0.17	-1.26	0.18

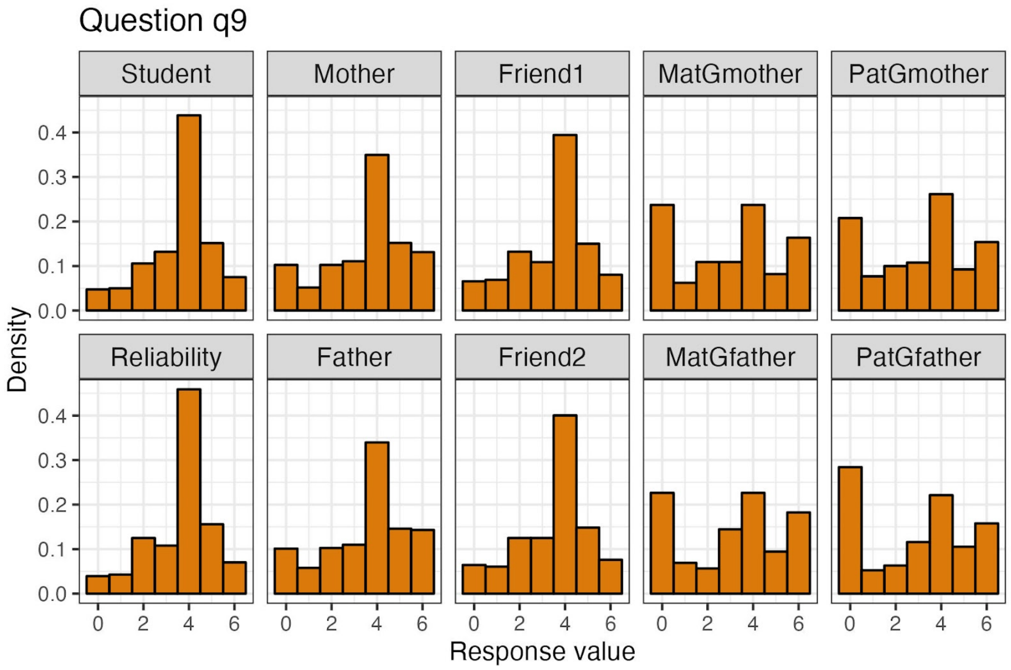

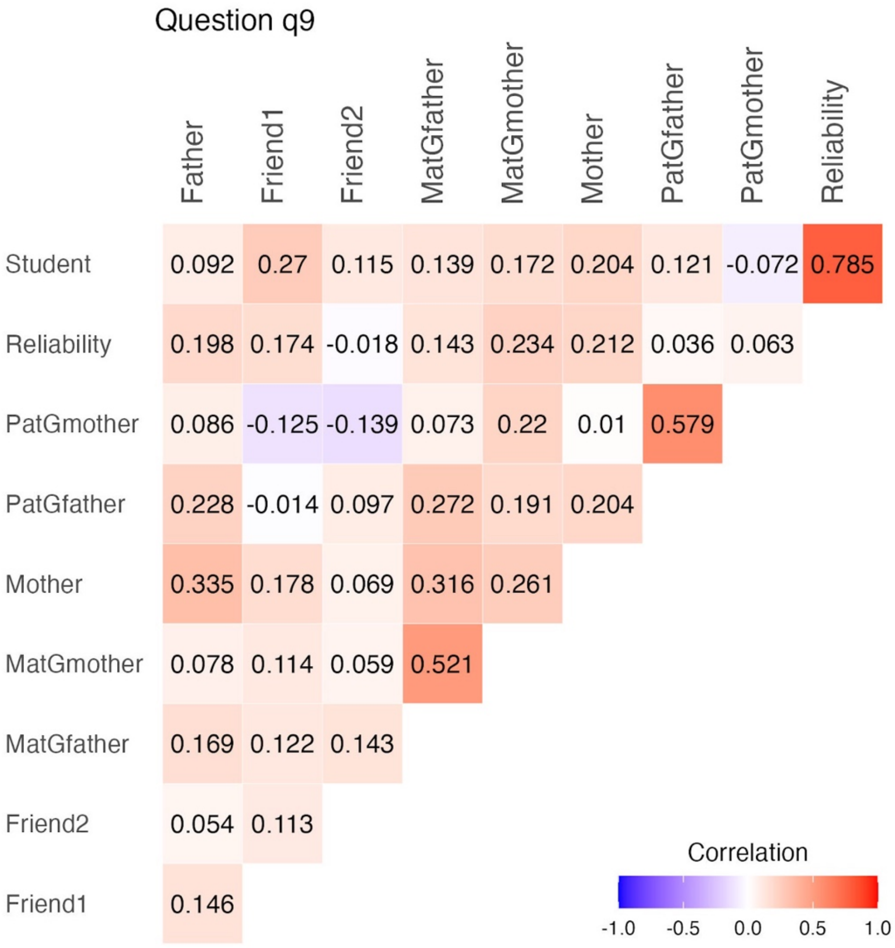

Question 10. How important is it to you to have a healthy lifestyle? Likert 0 to 6; higher values, more important.

	n	mean	sd	median	min	max	skew	kurtosis	se
Student	1901	4.61	1.19	5	0	6	-0.68	0.01	0.03
Reliability	584	4.70	1.08	5	1	6	-0.76	0.54	0.04
Friend1	1298	4.47	1.28	5	0	6	-0.76	0.33	0.04
Friend2	509	4.50	1.27	5	0	6	-0.80	0.52	0.06
Mother	832	5.03	1.15	5	0	6	-1.33	1.85	0.04
Father	691	4.77	1.23	5	0	6	-1.09	1.08	0.05
MatGfather	160	4.66	1.48	5	1	6	-0.95	-0.14	0.12
MatGmother	258	4.85	1.39	5	0	6	-1.22	0.97	0.09
PatGfather	94	4.71	1.43	5	0	6	-1.12	0.74	0.15
PatGmother	129	4.71	1.44	5	0	6	-0.93	0.07	0.13

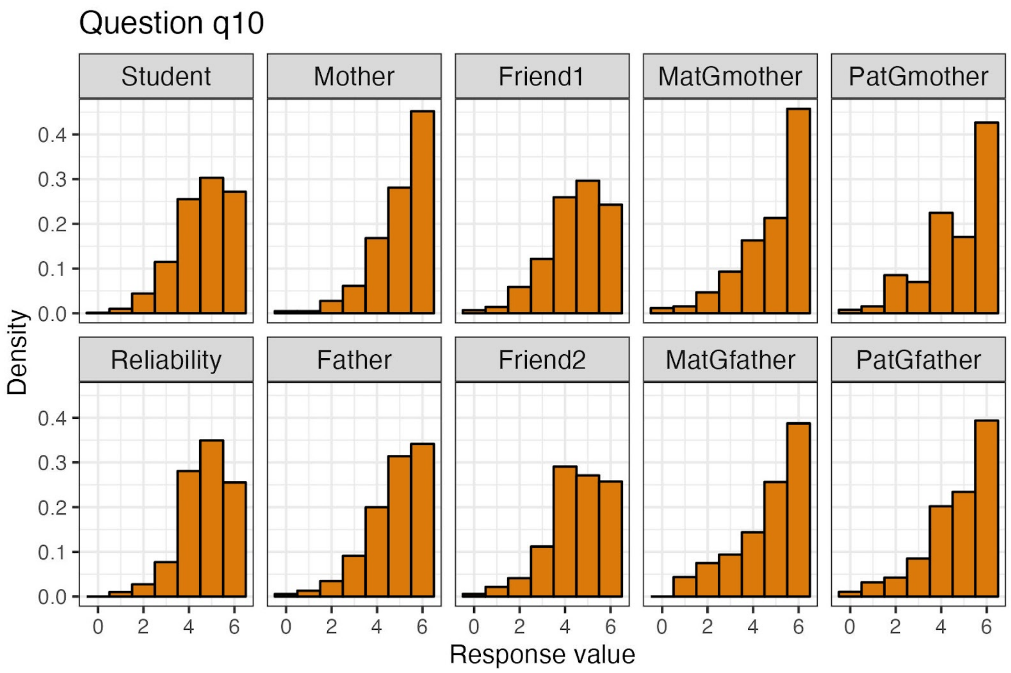

Question q10

	Father	Friend1	Friend2	MatGfather	MatGmother	Mother	PatGfather	PatGmother	Reliability
Student	0.11	0.195	0.104	0.137	0.073	0.118	-0.01	0.045	0.738
Reliability	0.145	0.187	0.014	0.024	0.049	0.154	-0.139	-0.29	
PatGmother	0.203	0.16	-0.019	0.105	0.571	0.254	0.323		
PatGfather	0.031	0.014	-0.156	0.426	0.127	0.024			
Mother	0.244	0.087	0.022	0.265	0.258				
MatGmother	0.16	0.281	-0.005	0.52					
MatGfather	0.109	0.129	0.11						
Friend2	-0.037	0.055							
Friend1	0.179								

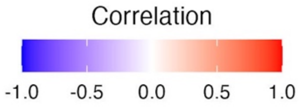

Question 11. What are your main forms of exercise? Tick all that apply. List of 15 & textbox for 'other'; Number of distinct forms of exercise.

	n	mean	sd	median	min	max	skew	kurtosis	se
Student	1905	2.95	1.48	3.0	0	15	1.31	4.44	0.03
Reliability	588	2.96	1.47	3.0	0	9	0.84	1.16	0.06
Friend1	1310	2.75	1.49	3.0	0	15	1.56	7.37	0.04
Friend2	512	2.79	1.49	3.0	1	10	1.14	2.23	0.07
Mother	835	1.96	1.09	2.0	0	6	1.01	0.68	0.04
Father	696	2.18	1.32	2.0	0	9	1.25	2.24	0.05
MatGfather	161	1.69	1.04	1.0	0	6	1.46	2.77	0.08
MatGmother	260	1.44	0.86	1.0	0	6	1.70	4.76	0.05
PatGfather	96	1.80	1.78	1.5	0	16	5.50	39.99	0.18
PatGmother	130	1.48	0.79	1.0	0	4	1.20	1.10	0.07

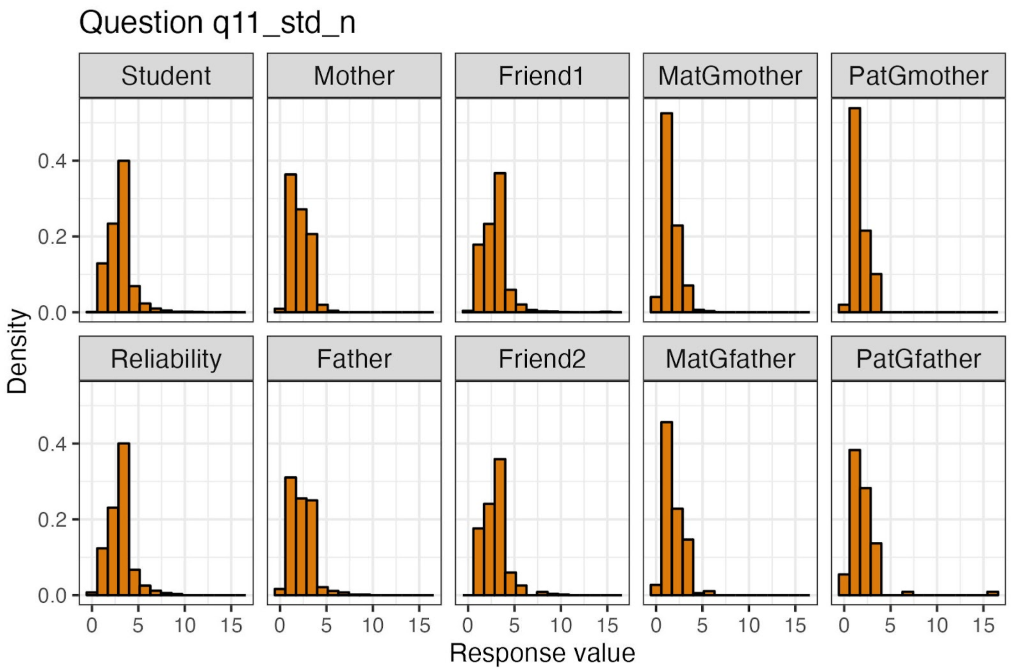

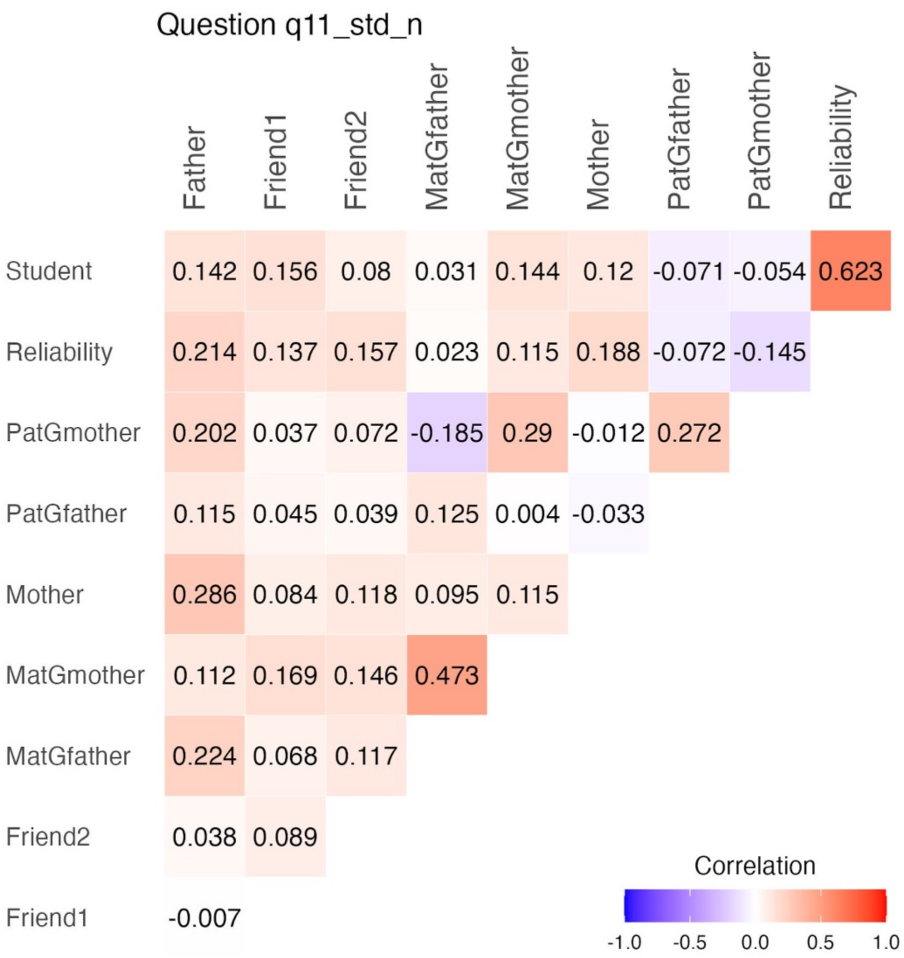

Question 12. How many hours of sleep do you get per night? Likert 0 to 6; higher values, more hours.

	n	mean	sd	median	min	max	skew	kurtosis	se
Student	1904	1.91	0.65	2	0	4	0.00	0.81	0.01
Reliability	584	1.91	0.65	2	0	4	0.09	0.89	0.03
Friend1	1309	1.88	0.65	2	0	4	-0.06	0.90	0.02
Friend2	512	1.81	0.66	2	0	4	-0.32	0.52	0.03
Mother	834	1.70	0.61	2	0	3	-0.37	0.20	0.02
Father	691	1.64	0.62	2	0	4	-0.11	0.05	0.02
MatGfather	160	1.93	0.77	2	0	4	0.21	0.27	0.06
MatGmother	258	1.89	0.77	2	0	4	0.14	0.09	0.05
PatGfather	96	1.98	0.78	2	0	4	0.43	0.34	0.08
PatGmother	130	1.92	0.73	2	0	4	0.24	-0.14	0.06

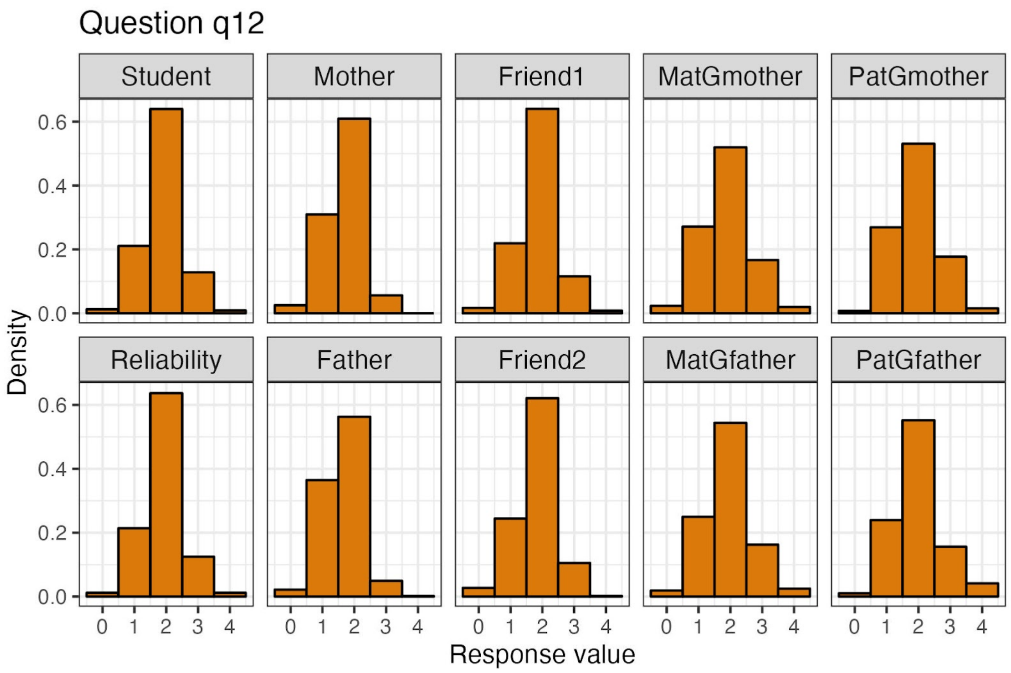
Question q12
Student
Mother
Friend1
MatGmother
PatGmother
Reliability
Father
Friend2
MatGfather
PatGfather
Density
0.0
0.2
0.4
0.6
0 1 2 3 4
Response value

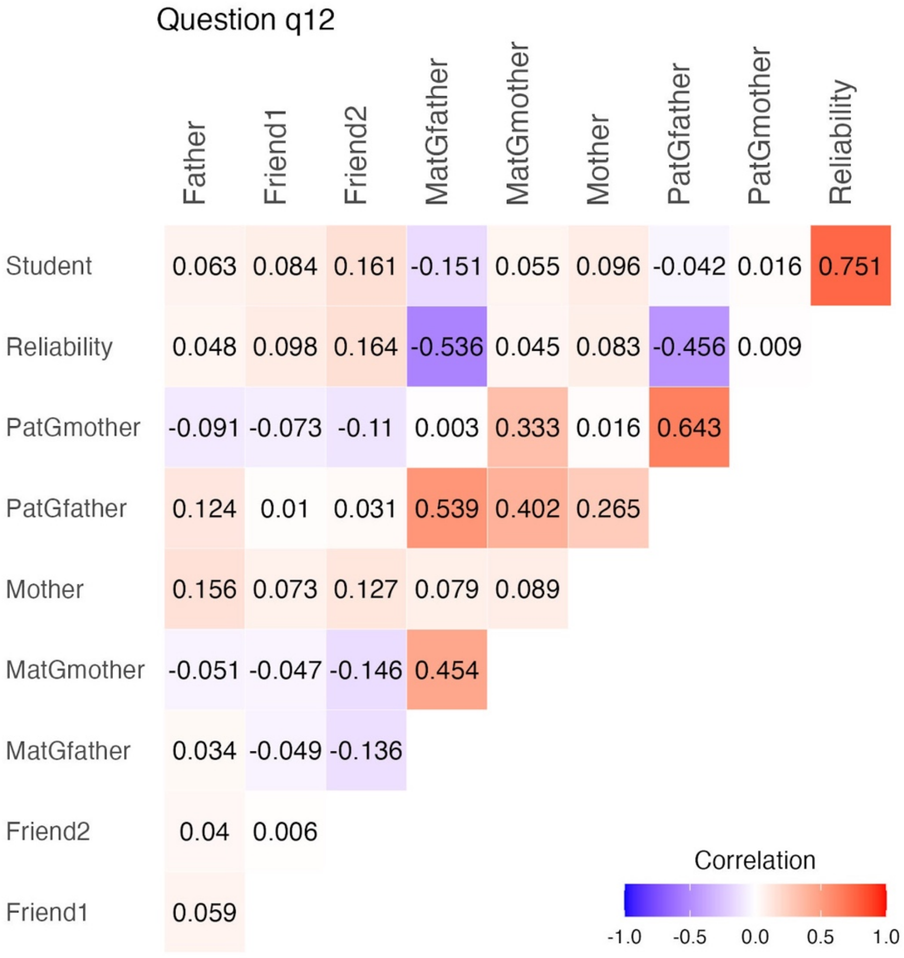
Question q12
Father
Friend1
Friend2
MatGfather
MatGmother
Mother
PatGfather
PatGmother
Reliability
Student 0.063 0.084 0.161 -0.151 0.055 0.096 -0.042 0.016 0.751
Reliability 0.048 0.098 0.164 -0.536 0.045 0.083 -0.456 0.009
PatGmother -0.091 -0.073 -0.11 0.003 0.333 0.016 0.643
PatGfather 0.124 0.01 0.031 0.539 0.402 0.265
Mother 0.156 0.073 0.127 0.079 0.089
MatGmother -0.051 -0.047 -0.146 0.454
MatGfather 0.034 -0.049 -0.136
Friend2 0.04 0.006
Friend1 0.059
Correlation
-1.0 -0.5 0.0 0.5 1.0

Question 13. How many cigarettes do you smoke per day? Likert: 0 to 6; higher values, more cigarettes.

	n	mean	sd	median	min	max	skew	kurtosis	se
Student	1902	0.07	0.34	0	0	4	5.69	40.10	0.01
Reliability	583	0.06	0.28	0	0	2	4.83	24.66	0.01
Friend1	1308	0.08	0.40	0	0	4	6.13	43.66	0.01
Friend2	512	0.11	0.41	0	0	4	4.89	29.22	0.02
Mother	831	0.08	0.46	0	0	4	6.20	39.62	0.02
Father	691	0.21	0.67	0	0	4	3.43	11.37	0.03
MatGfather	160	0.19	0.63	0	0	4	3.74	14.64	0.05
MatGmother	256	0.11	0.48	0	0	4	5.04	28.06	0.03
PatGfather	95	0.22	0.67	0	0	4	3.48	12.86	0.07
PatGmother	129	0.08	0.43	0	0	4	7.07	56.64	0.04

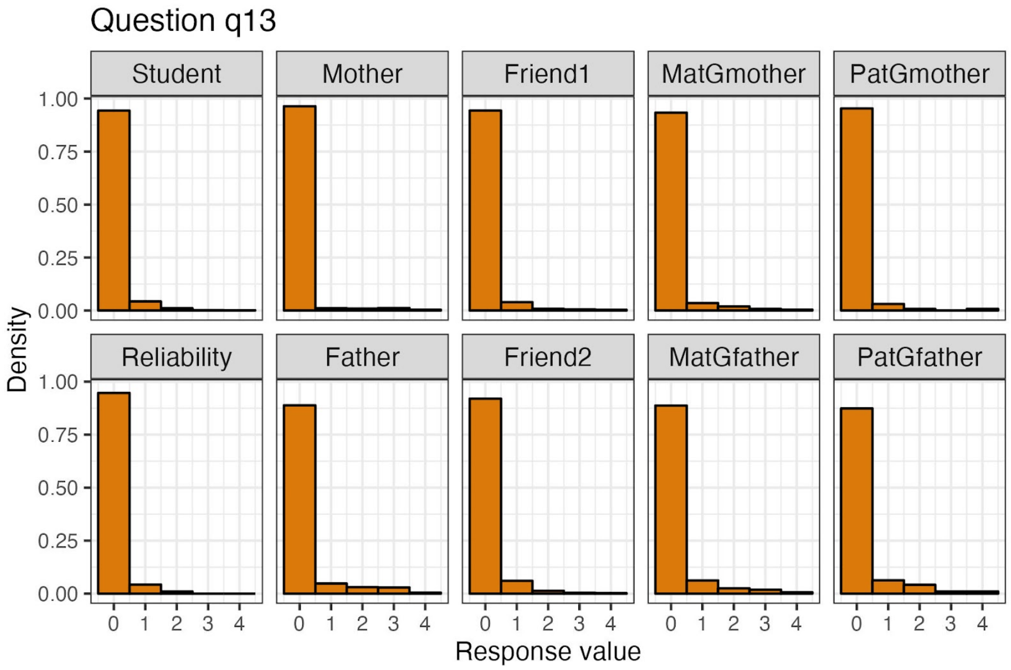

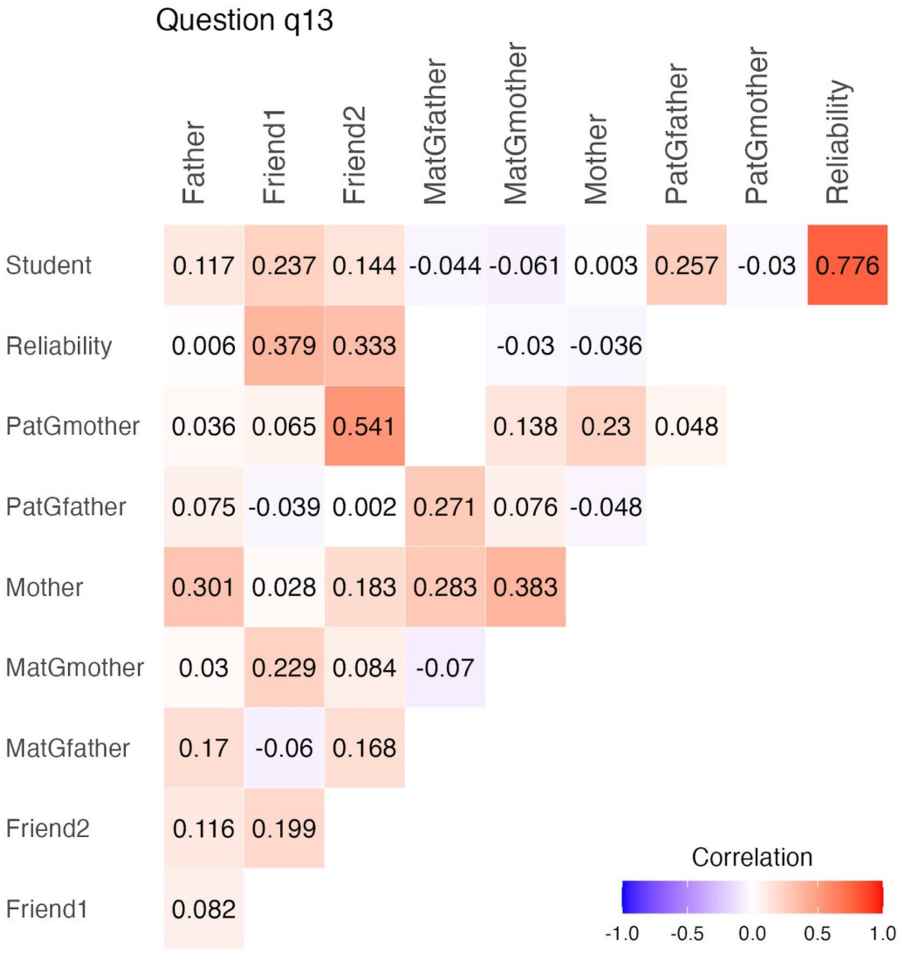

Question 14. How many alcoholic drinks do you consume per week? Likert 0 to 6; higher values, more drinks.

	n	mean	sd	median	min	max	skew	kurtosis	se
Student	1899	1.07	1.10	1	0	4	0.80	-0.24	0.03
Reliability	587	1.09	1.05	1	0	4	0.74	-0.29	0.04
Friend1	1306	1.06	1.13	1	0	4	0.85	-0.19	0.03
Friend2	511	1.17	1.15	1	0	4	0.66	-0.62	0.05
Mother	834	1.00	1.12	1	0	4	0.81	-0.43	0.04
Father	691	1.52	1.31	1	0	4	0.41	-1.00	0.05
MatGfather	160	1.24	1.27	1	0	4	0.67	-0.69	0.10
MatGmother	258	0.72	1.02	0	0	4	1.29	0.70	0.06
PatGfather	95	1.25	1.30	1	0	4	0.60	-0.98	0.13
PatGmother	130	0.69	1.02	0	0	3	1.20	0.07	0.09

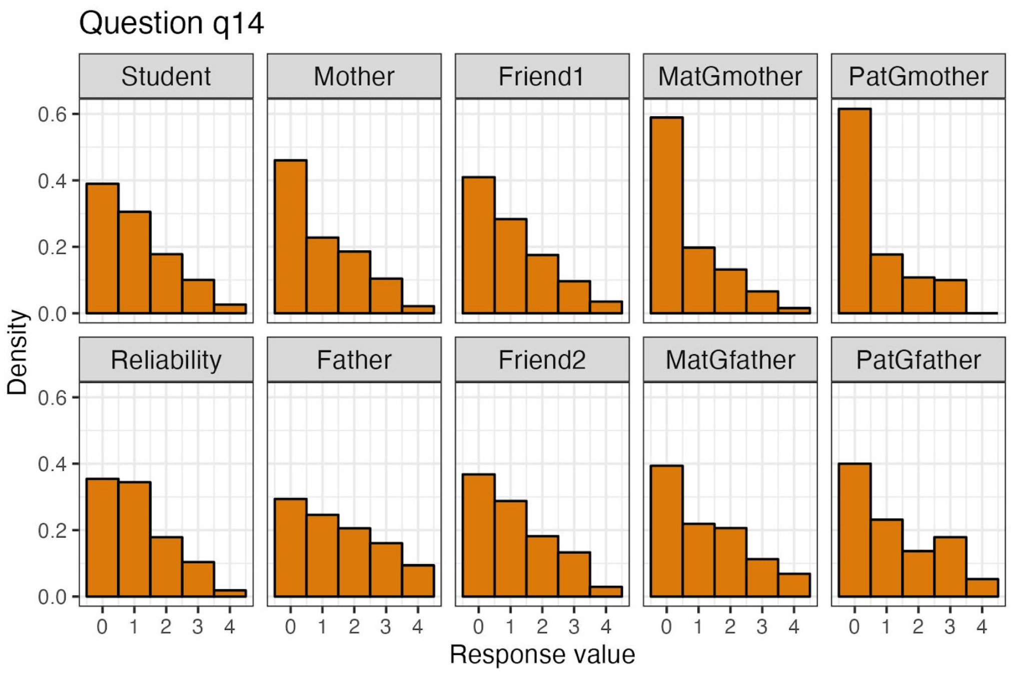
Question q14
Student
Mother
Friend1
MatGmother
PatGmother
Reliability
Father
Friend2
MatGfather
PatGfather
Density
0.0
0.2
0.4
0.6
0 1 2 3 4
Response value

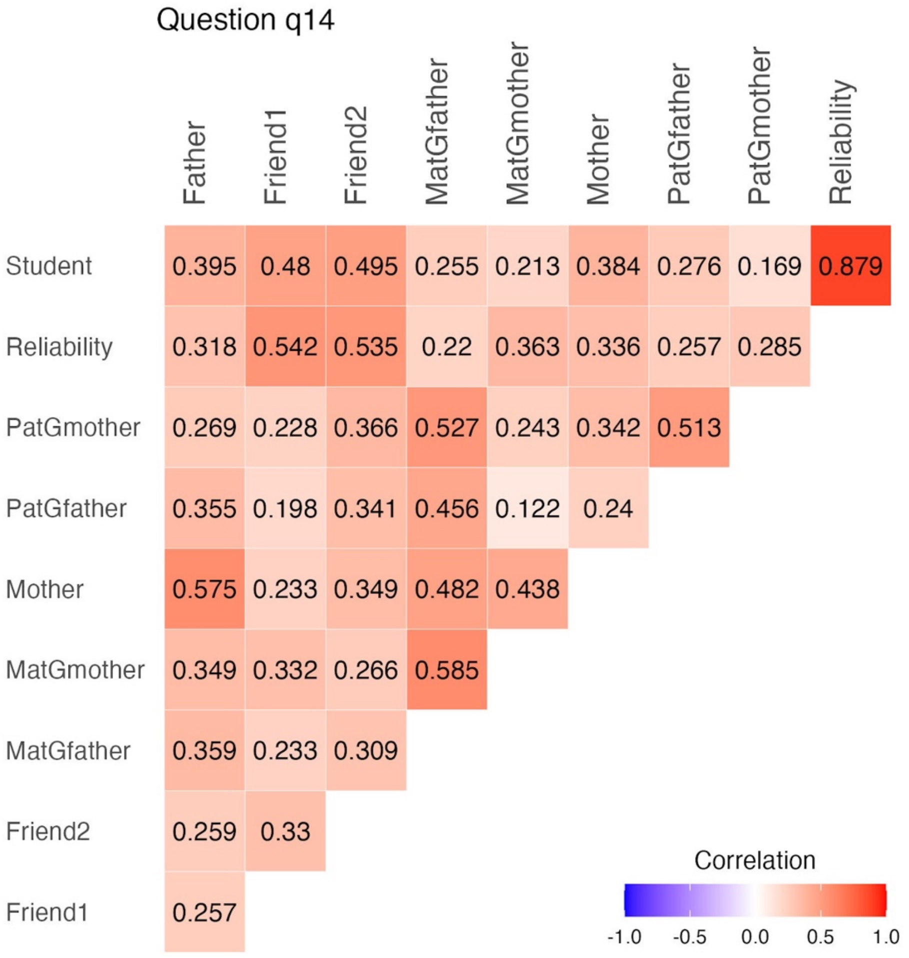
Question q14
Father
Friend1
Friend2
MatGfather
MatGmother
Mother
PatGfather
PatGmother
Reliability
Student 0.395 0.48 0.495 0.255 0.213 0.384 0.276 0.169 0.879
Reliability 0.318 0.542 0.535 0.22 0.363 0.336 0.257 0.285
PatGmother 0.269 0.228 0.366 0.527 0.243 0.342 0.513
PatGfather 0.355 0.198 0.341 0.456 0.122 0.24
Mother 0.575 0.233 0.349 0.482 0.438
MatGmother 0.349 0.332 0.266 0.585
MatGfather 0.359 0.233 0.309
Friend2 0.259 0.33
Friend1 0.257
Correlation
-1.0
-0.5
0.0
0.5
1.0

Question 15. How would you describe your diet? Tick all that apply. Recoded: 0=vegan, 1=vegetarian, 2=pescatarian, 3=flexitarian, 4=no restrictions, 5=no red meat, 6=mostly meat-eater

	n	mean	sd	median	min	max	skew	kurtosis	se
Student	1897	3.64	1.00	4	0	6	-2.62	5.63	0.02
Reliability	586	3.64	0.98	4	0	5	-2.54	5.21	0.04
Friend1	1308	3.66	0.99	4	0	6	-2.61	5.74	0.03
Friend2	512	3.65	1.01	4	0	5	-2.75	6.18	0.04
Mother	834	3.71	0.87	4	0	5	-2.93	7.28	0.03
Father	691	3.79	0.77	4	0	6	-3.52	11.79	0.03
MatGfather	157	3.94	0.50	4	1	6	-4.65	28.56	0.04
MatGmother	254	3.78	0.78	4	0	4	-3.34	9.57	0.05
PatGfather	96	3.81	0.73	4	0	4	-3.88	13.97	0.07
PatGmother	130	3.74	0.85	4	1	5	-2.79	6.19	0.07

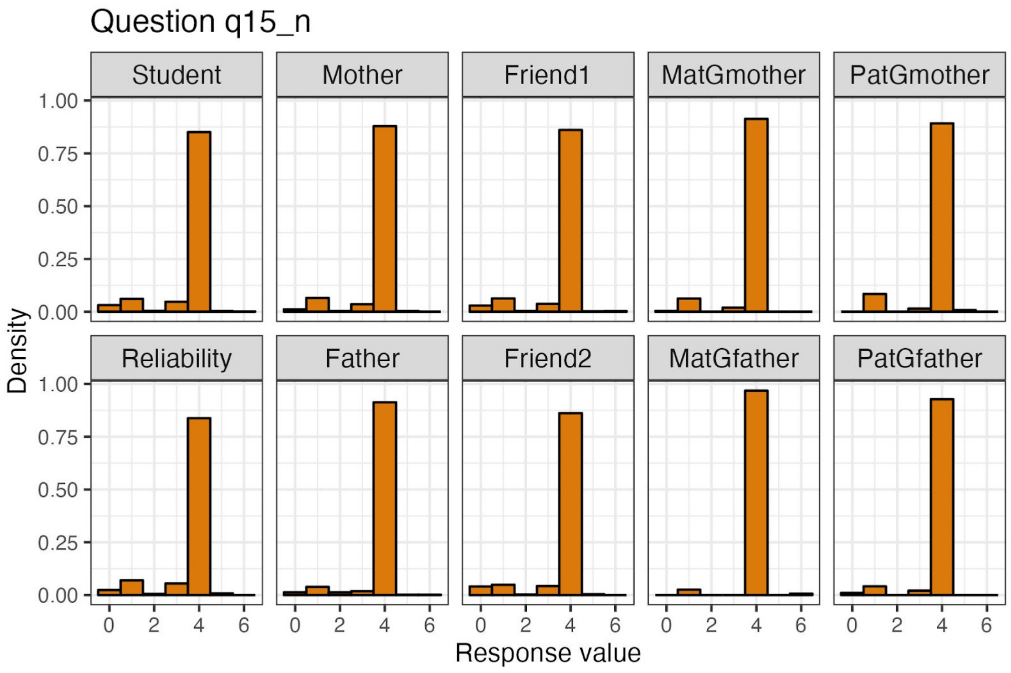

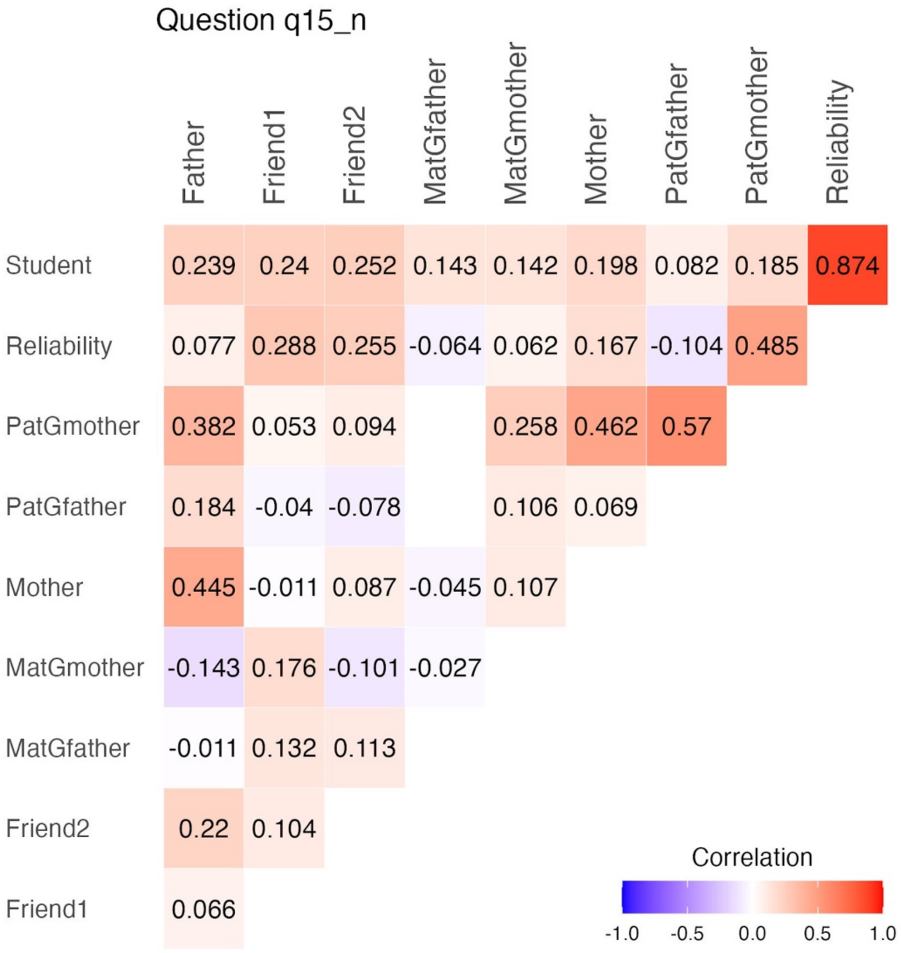

Question 16 How often do you eat a serve of fresh fruit or vegetables? Tick the one option that is most true for you. Likert 0 to 6; higher values, more

often.

	n	mean	sd	median	min	max	skew	kurtosis	se
Student	1905	4.23	1.30	5	0	6	-0.66	-0.22	0.03
Reliability	586	4.22	1.31	5	0	6	-0.73	0.08	0.05
Friend1	1309	4.09	1.36	4	0	6	-0.62	-0.13	0.04
Friend2	511	4.10	1.41	5	0	6	-0.62	-0.40	0.06
Mother	835	4.73	1.08	5	0	6	-0.89	0.48	0.04
Father	693	4.25	1.22	5	0	6	-0.66	0.07	0.05
MatGfather	160	4.35	1.33	5	1	6	-0.86	0.00	0.10
MatGmother	258	4.56	1.22	5	0	6	-0.99	0.89	0.08
PatGfather	96	4.41	1.17	5	0	6	-0.82	0.96	0.12
PatGmother	129	4.40	1.30	5	0	6	-1.04	0.91	0.11

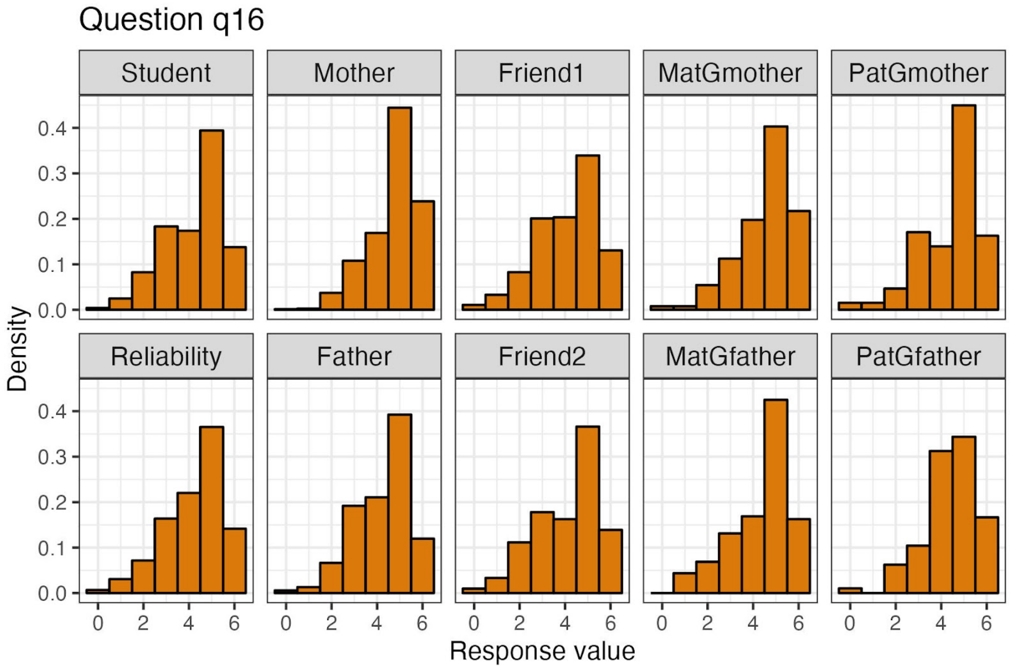

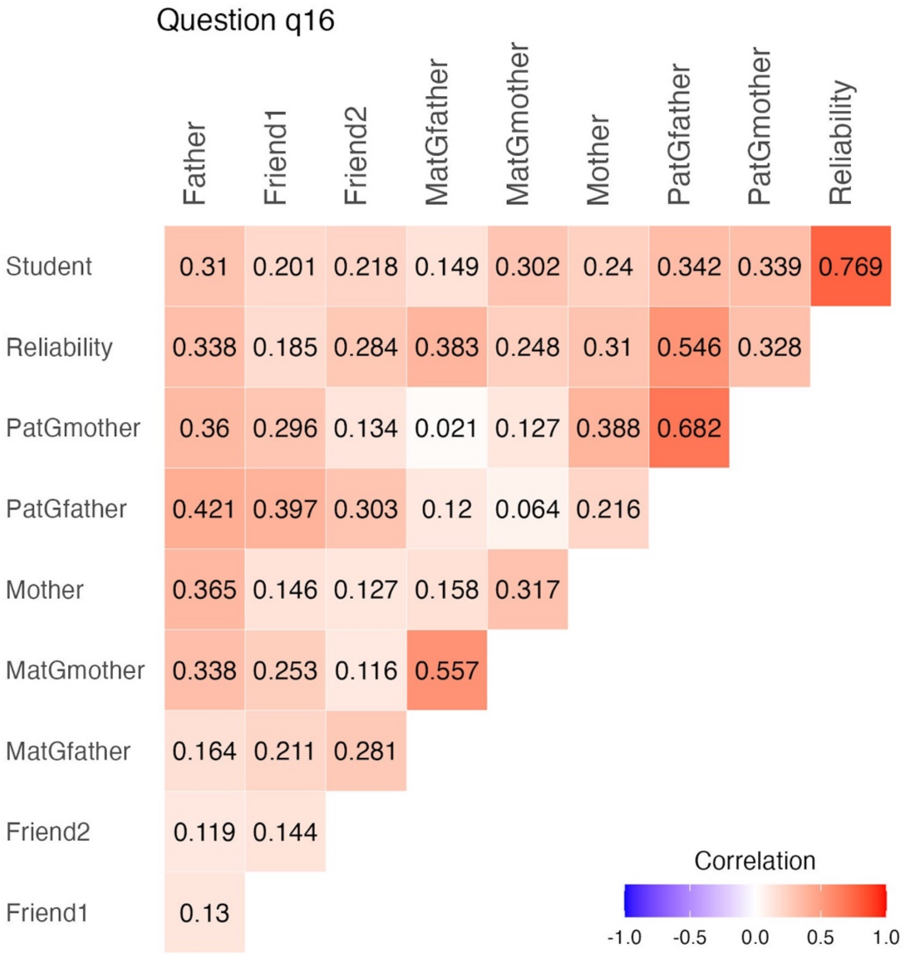

Question 17. How many hours do you spend watching TV/movies/videos (including online)? Likert 0 to 6; higher values, more hours.

	n	mean	sd	median	min	max	skew	kurtosis	se
Student	1898	3.41	1.14	3	0	6	-0.18	0.04	0.03
Reliability	585	3.45	1.20	4	0	6	-0.19	-0.19	0.05
Friend1	1309	3.51	1.19	4	0	6	-0.24	0.13	0.03
Friend2	512	3.46	1.19	4	0	6	-0.24	0.18	0.05
Mother	833	2.92	1.09	3	0	6	-0.19	0.09	0.04
Father	689	2.95	1.05	3	0	6	-0.32	0.20	0.04
MatGfather	158	3.23	1.30	3	0	6	-0.65	0.12	0.10
MatGmother	255	3.25	1.28	4	0	6	-0.43	-0.31	0.08
PatGfather	96	3.26	1.34	4	0	6	-0.50	-0.28	0.14
PatGmother	130	3.31	1.26	4	0	6	-0.45	0.04	0.11

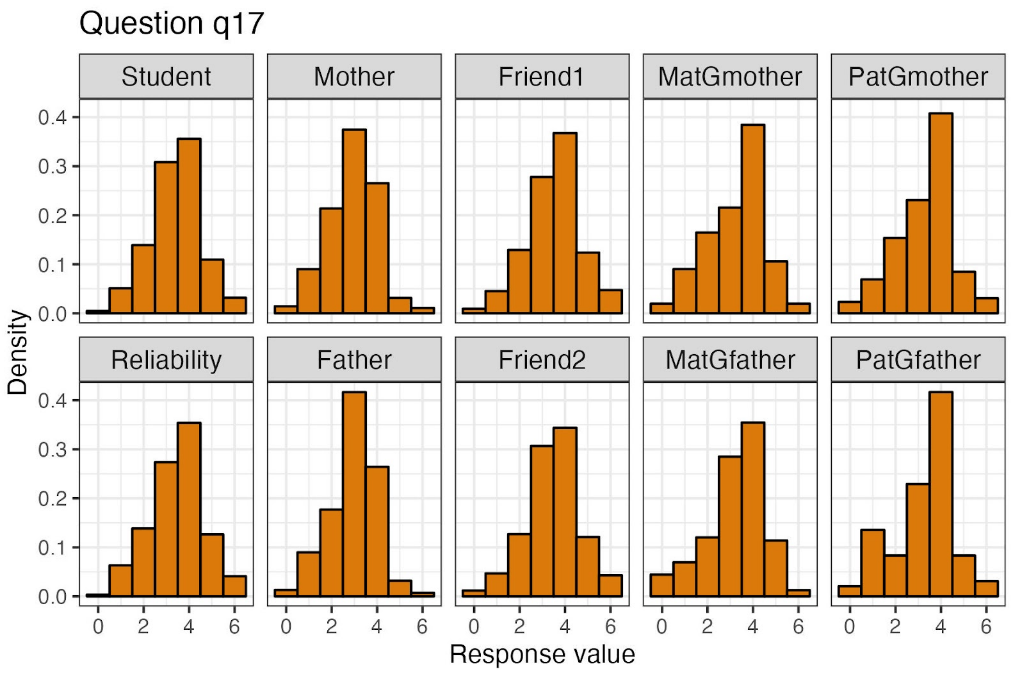
Question q17
Student
Mother
Friend1
MatGmother
PatGmother
Reliability
Father
Friend2
MatGfather
PatGfather
Density
0.0
0.1
0.2
0.3
0.4
0
2
4
6
Response value

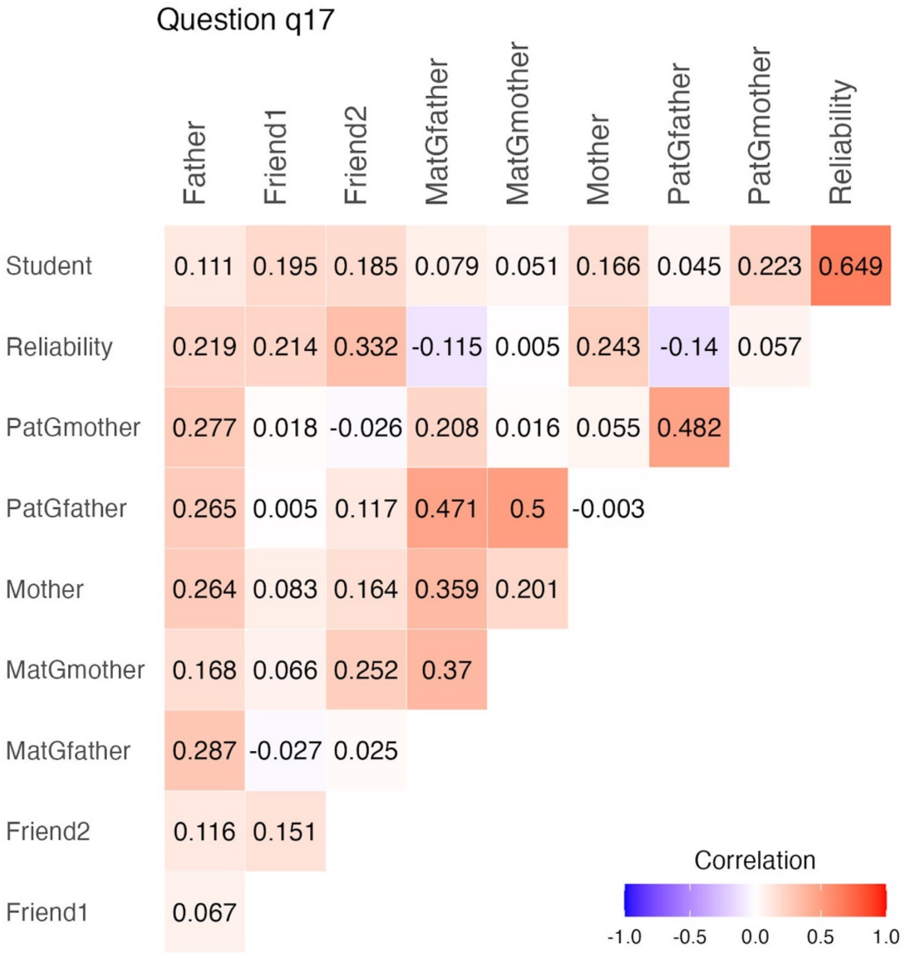
Question q17
Father
Friend1
Friend2
MatGfather
MatGmother
Mother
PatGfather
PatGmother
Reliability
Student 0.111 0.195 0.185 0.079 0.051 0.166 0.045 0.223 0.649
Reliability 0.219 0.214 0.332 -0.115 0.005 0.243 -0.14 0.057
PatGmother 0.277 0.018 -0.026 0.208 0.016 0.055 0.482
PatGfather 0.265 0.005 0.117 0.471 0.5 -0.003
Mother 0.264 0.083 0.164 0.359 0.201
MatGmother 0.168 0.066 0.252 0.37
MatGfather 0.287 -0.027 0.025
Friend2 0.116 0.151
Friend1 0.067
Correlation
-1.0
-0.5
0.0
0.5
1.0

Question 18. How much do you enjoy watching TV/movies/videos (including online)? Likert 0 to 6; higher values, more enjoyment.

	n	mean	sd	median	min	max	skew	kurtosis	se
Student	1903	4.72	1.21	5	0	6	-0.99	0.76	0.03
Reliability	587	4.72	1.19	5	0	6	-1.00	1.14	0.05
Friend1	1306	4.64	1.24	5	0	6	-0.96	0.98	0.03
Friend2	510	4.63	1.27	5	0	6	-1.00	0.98	0.06
Mother	834	4.06	1.43	4	0	6	-0.47	-0.31	0.05
Father	689	4.03	1.35	4	0	6	-0.60	0.23	0.05
MatGfather	160	3.98	1.62	4	0	6	-0.71	-0.12	0.13
MatGmother	257	3.89	1.64	4	0	6	-0.45	-0.54	0.10
PatGfather	95	4.02	1.56	4	0	6	-0.70	0.02	0.16
PatGmother	130	3.95	1.50	4	0	6	-0.61	-0.09	0.13

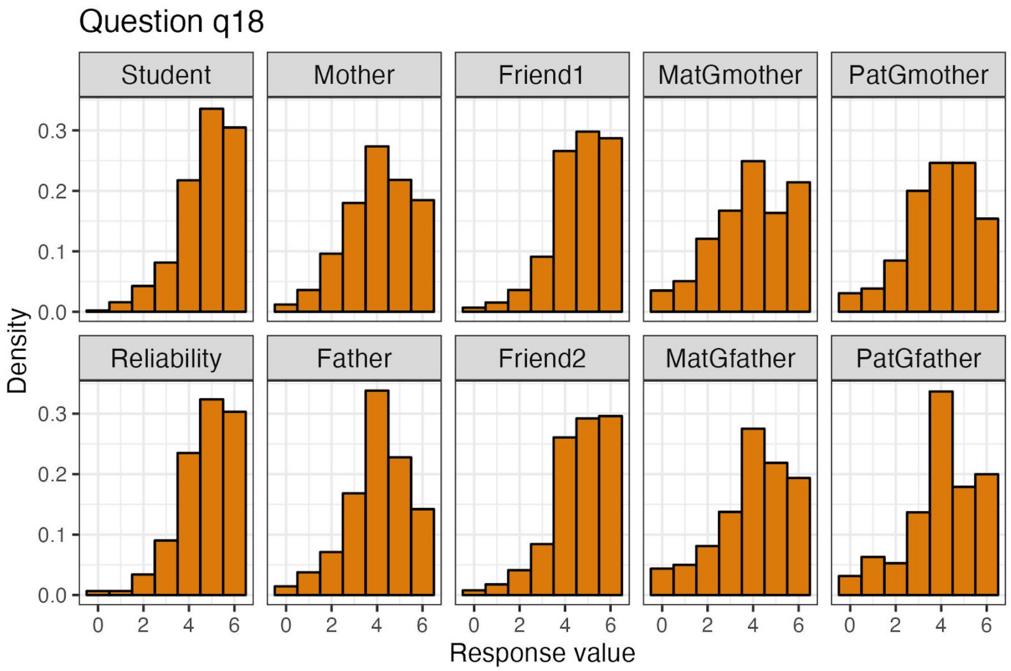

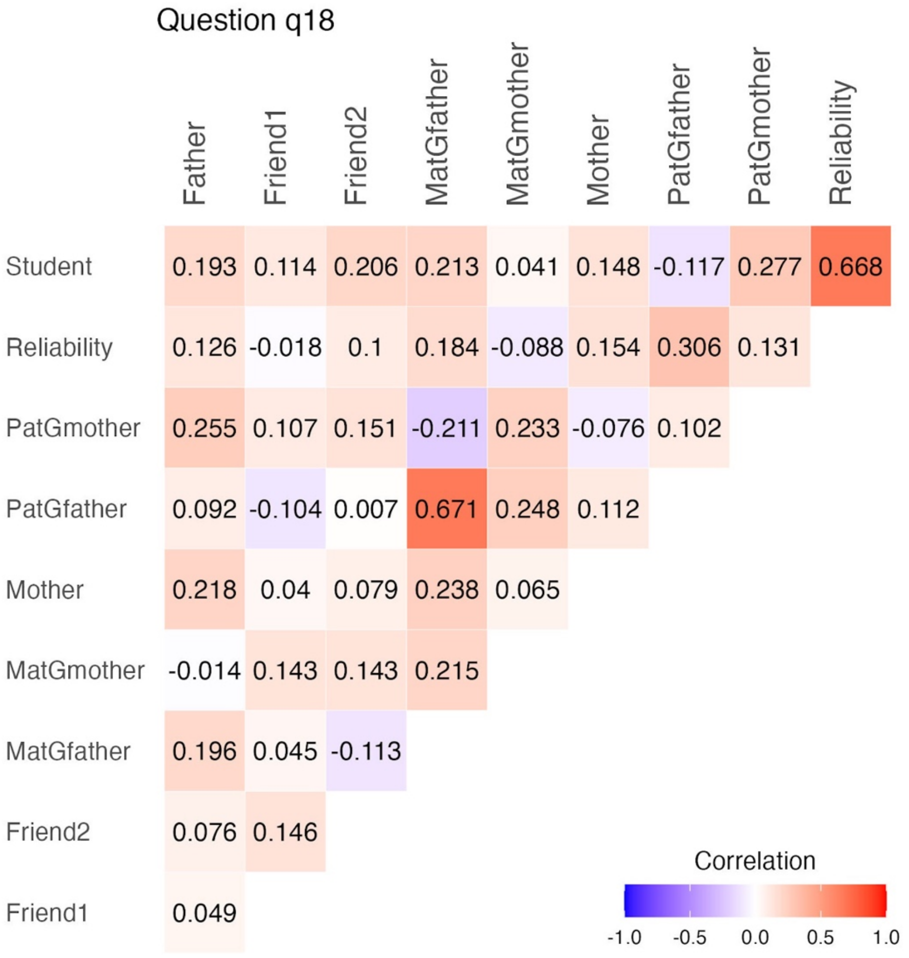

Question 19. How many hours do you spend reading for pleasure? Likert 0 to 6; higher values, more hours.

	n	mean	sd	median	min	max	skew	kurtosis	se
Student	1903	1.34	1.18	1	0	6	0.87	0.52	0.03
Reliability	587	1.41	1.22	1	0	6	0.82	0.34	0.05
Friend1	1310	1.25	1.24	1	0	6	0.96	0.48	0.03
Friend2	511	1.34	1.31	1	0	6	0.98	0.56	0.06
Mother	834	1.73	1.16	2	0	5	0.23	-0.79	0.04
Father	691	1.60	1.20	1	0	6	0.42	-0.40	0.05
MatGfather	159	1.99	1.47	2	0	6	0.33	-0.67	0.12
MatGmother	258	2.17	1.39	2	0	6	-0.14	-0.90	0.09
PatGfather	96	2.19	1.35	2	0	6	0.09	-0.68	0.14
PatGmother	130	2.26	1.43	3	0	6	-0.10	-0.90	0.13

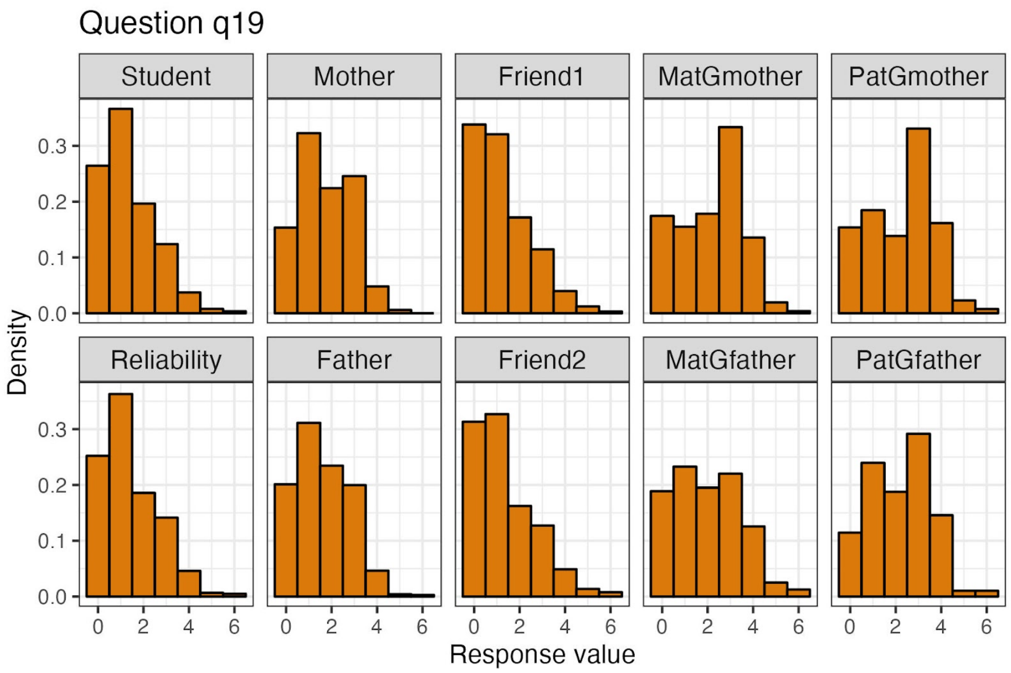
Question q19
Student
Mother
Friend1
MatGmother
PatGmother
Reliability
Father
Friend2
MatGfather
PatGfather
Density
0.0
0.1
0.2
0.3
0
2
4
6
Response value

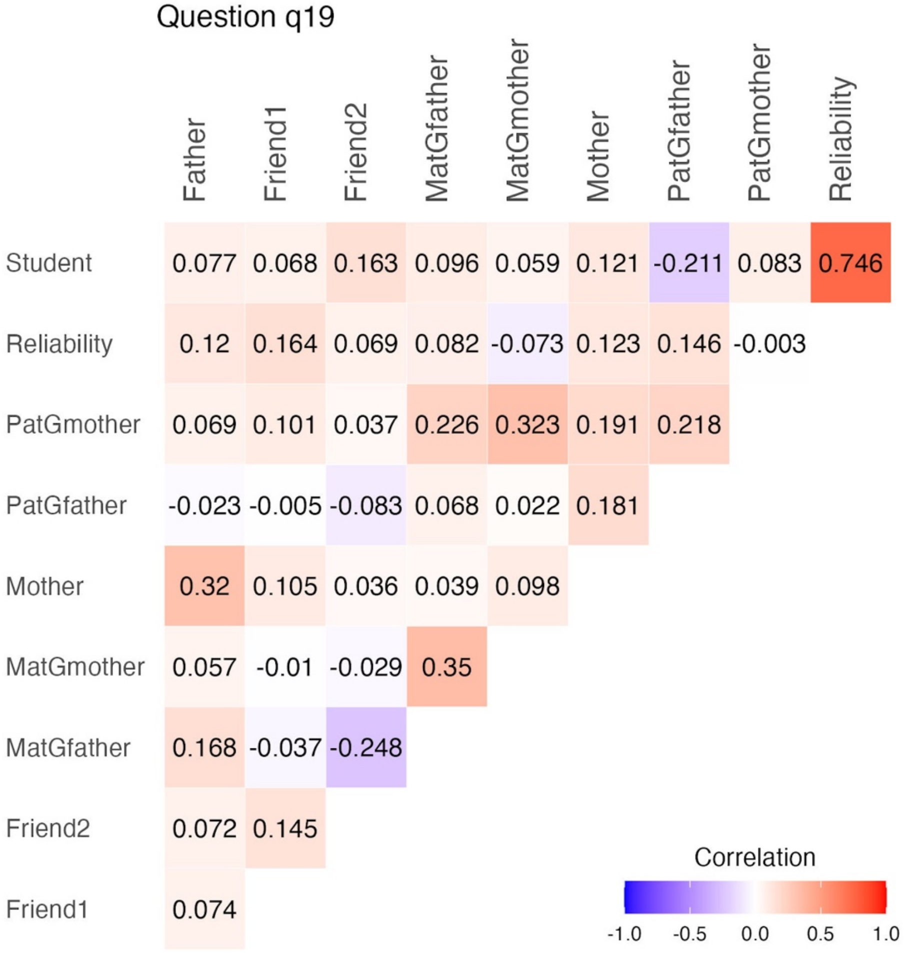
Question q19
Father
Friend1
Friend2
MatGfather
MatGmother
Mother
PatGfather
PatGmother
Reliability
Student 0.077 0.068 0.163 0.096 0.059 0.121 -0.211 0.083 0.746
Reliability 0.12 0.164 0.069 0.082 -0.073 0.123 0.146 -0.003
PatGmother 0.069 0.101 0.037 0.226 0.323 0.191 0.218
PatGfather -0.023 -0.005 -0.083 0.068 0.022 0.181
Mother 0.32 0.105 0.036 0.039 0.098
MatGmother 0.057 -0.01 -0.029 0.35
MatGfather 0.168 -0.037 -0.248
Friend2 0.072 0.145
Friend1 0.074
Correlation
-1.0
-0.5
0.0
0.5
1.0

Question 20. How much do you enjoy reading? Likert 0 to 6; higher values, more enjoyment.

	n	mean	sd	median	min	max	skew	kurtosis	se
Student	1902	4.08	1.65	4	0	6	-0.71	-0.29	0.04
Reliability	586	4.20	1.63	5	0	6	-0.85	-0.06	0.07
Friend1	1307	3.61	1.87	4	0	6	-0.41	-0.90	0.05
Friend2	511	3.69	1.86	4	0	6	-0.50	-0.76	0.08
Mother	833	4.30	1.65	5	0	6	-0.89	0.11	0.06
Father	691	3.74	1.75	4	0	6	-0.56	-0.55	0.07
MatGfather	158	3.90	1.81	4	0	6	-0.73	-0.40	0.14
MatGmother	257	4.21	1.83	5	0	6	-0.89	-0.25	0.11
PatGfather	95	3.87	1.83	4	0	6	-0.52	-0.71	0.19
PatGmother	129	4.20	1.97	5	0	6	-0.87	-0.50	0.17

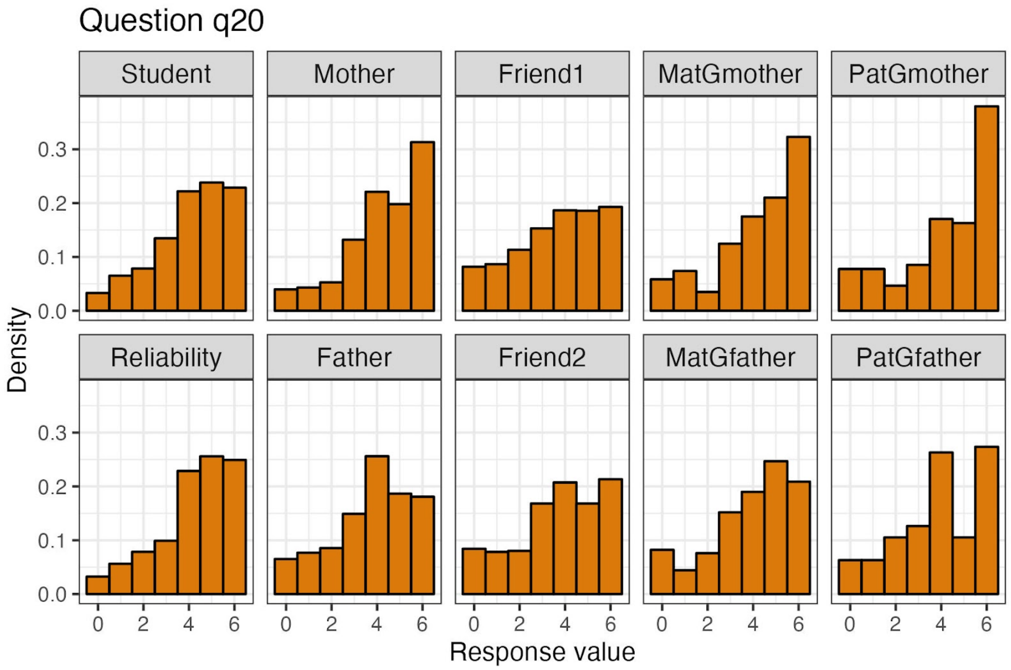

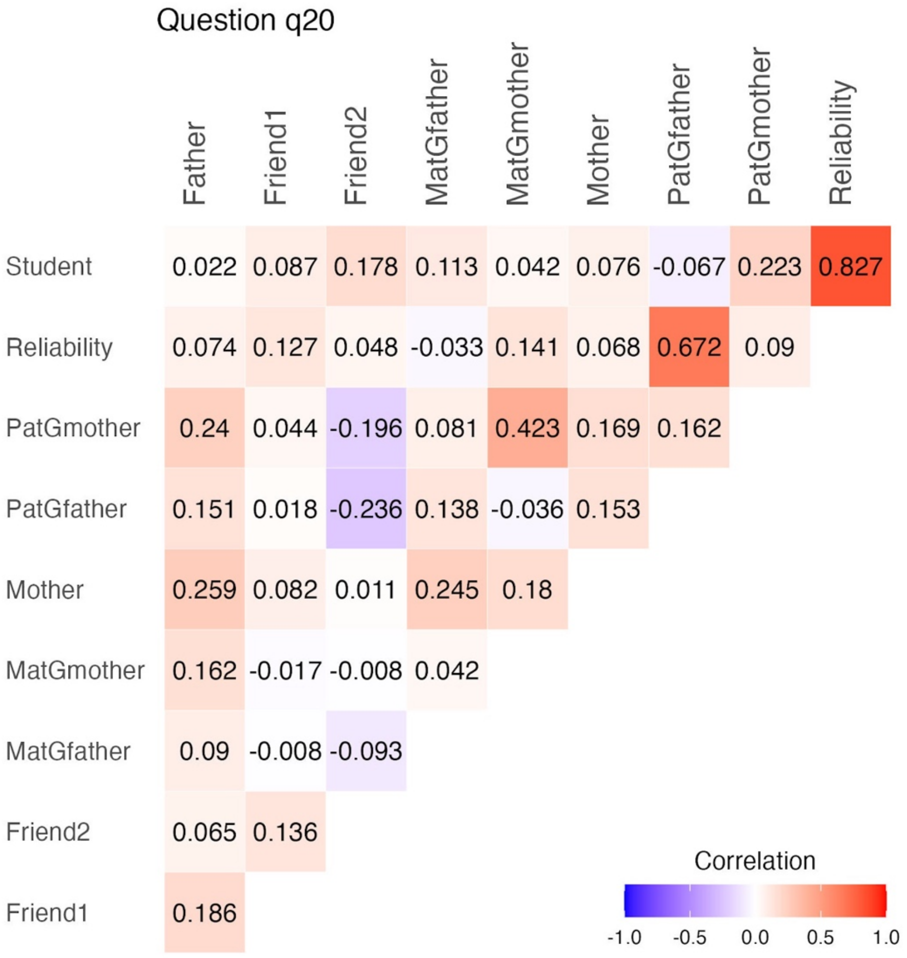

Question 21. How many hours do you spend listening to music? Likert 0 to 6; higher values, more hours.

	n	mean	sd	median	min	max	skew	kurtosis	se
Student	1902	4.08	1.65	4	0	6	-0.71	-0.29	0.04
Reliability	586	4.20	1.63	5	0	6	-0.85	-0.06	0.07
Friend1	1307	3.61	1.87	4	0	6	-0.41	-0.90	0.05
Friend2	511	3.69	1.86	4	0	6	-0.50	-0.76	0.08
Mother	833	4.30	1.65	5	0	6	-0.89	0.11	0.06
Father	691	3.74	1.75	4	0	6	-0.56	-0.55	0.07
MatGfather	158	3.90	1.81	4	0	6	-0.73	-0.40	0.14
MatGmother	257	4.21	1.83	5	0	6	-0.89	-0.25	0.11
PatGfather	95	3.87	1.83	4	0	6	-0.52	-0.71	0.19
PatGmother	129	4.20	1.97	5	0	6	-0.87	-0.50	0.17

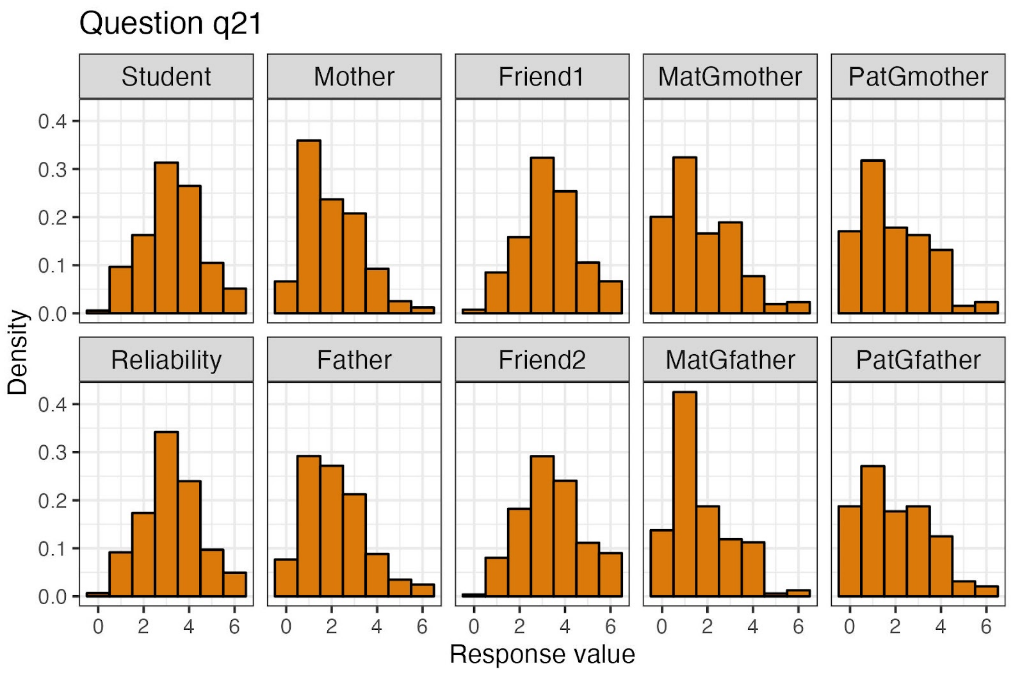
Question q21
Student
Mother
Friend1
MatGmother
PatGmother
Reliability
Father
Friend2
MatGfather
PatGfather
Density
0.0
0.1
0.2
0.3
0.4
0 2 4 6
Response value

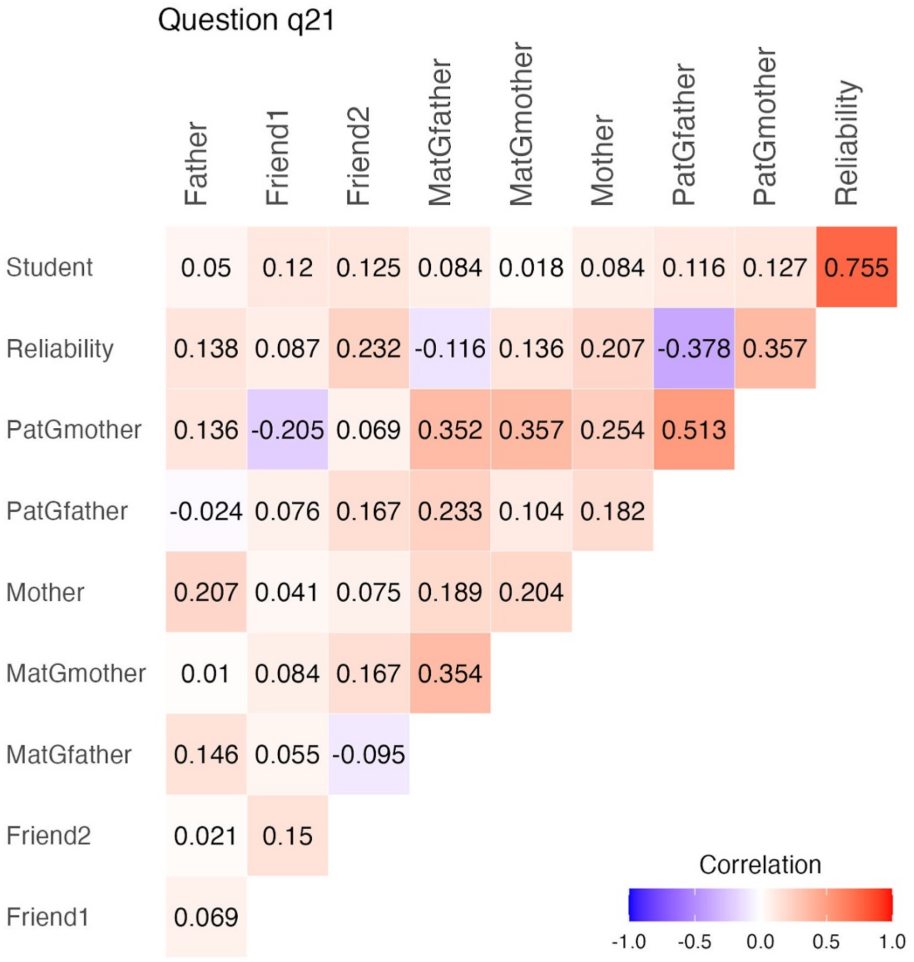
Question q21
Father
Friend1
Friend2
MatGfather
MatGmother
Mother
PatGfather
PatGmother
Reliability
Student 0.05 0.12 0.125 0.084 0.018 0.084 0.116 0.127 0.755
Reliability 0.138 0.087 0.232 -0.116 0.136 0.207 -0.378 0.357
PatGmother 0.136 -0.205 0.069 0.352 0.357 0.254 0.513
PatGfather -0.024 0.076 0.167 0.233 0.104 0.182
Mother 0.207 0.041 0.075 0.189 0.204
MatGmother 0.01 0.084 0.167 0.354
MatGfather 0.146 0.055 -0.095
Friend2 0.021 0.15
Friend1 0.069
Correlation
-1.0 -0.5 0.0 0.5 1.0

Question 22. How much do you enjoy listening to music? Likert 0 to 6; higher values, more enjoyment.

	n	mean	sd	median	min	max	skew	kurtosis	se
Student	1903	5.46	0.87	6	0	6	-1.93	4.66	0.02
Reliability	588	5.39	0.91	6	1	6	-1.70	3.03	0.04
Friend1	1305	5.37	0.97	6	0	6	-1.87	4.19	0.03
Friend2	510	5.34	0.97	6	1	6	-1.74	3.21	0.04
Mother	833	4.64	1.29	5	0	6	-0.72	-0.03	0.04
Father	692	4.48	1.44	5	0	6	-0.96	0.46	0.05
MatGfather	159	4.01	1.65	4	0	6	-0.55	-0.53	0.13
MatGmother	259	4.02	1.75	4	0	6	-0.62	-0.47	0.11
PatGfather	95	3.85	1.77	4	0	6	-0.53	-0.50	0.18
PatGmother	129	3.86	1.88	4	0	6	-0.59	-0.71	0.17

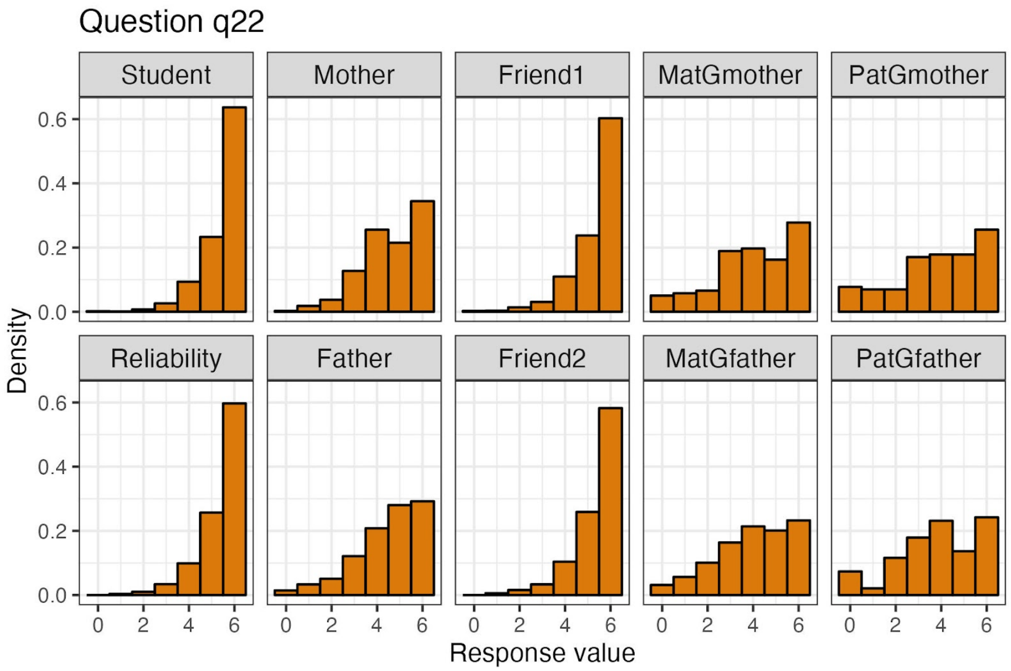

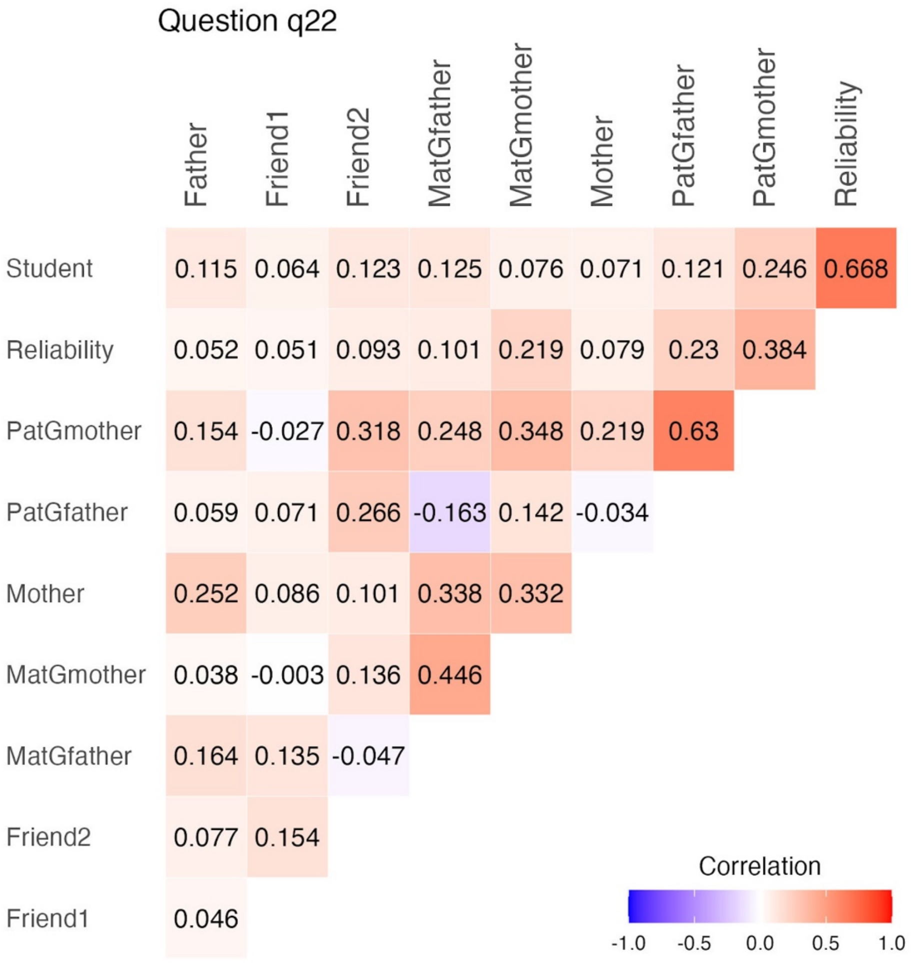

Question 23. How many close friends do you have? Likert 0 to 6; higher values, more close friends.

	n	mean	sd	median	min	max	skew	kurtosis	se
Student	1903	2.66	1.34	2	0	6	0.82	0.29	0.03
Reliability	587	2.56	1.25	2	0	6	0.84	0.72	0.05
Friend1	1308	2.96	1.36	3	0	6	0.64	-0.03	0.04
Friend2	511	3.01	1.38	3	1	6	0.69	-0.23	0.06
Mother	830	2.72	1.50	2	0	6	0.81	-0.01	0.05
Father	687	2.57	1.55	2	0	6	0.75	-0.09	0.06
MatGfather	160	2.61	1.77	2	0	6	0.65	-0.59	0.14
MatGmother	252	2.67	1.52	2	0	6	0.69	-0.09	0.10
PatGfather	94	2.64	1.69	2	0	6	0.69	-0.43	0.17
PatGmother	129	2.63	1.57	2	0	6	0.56	-0.42	0.14

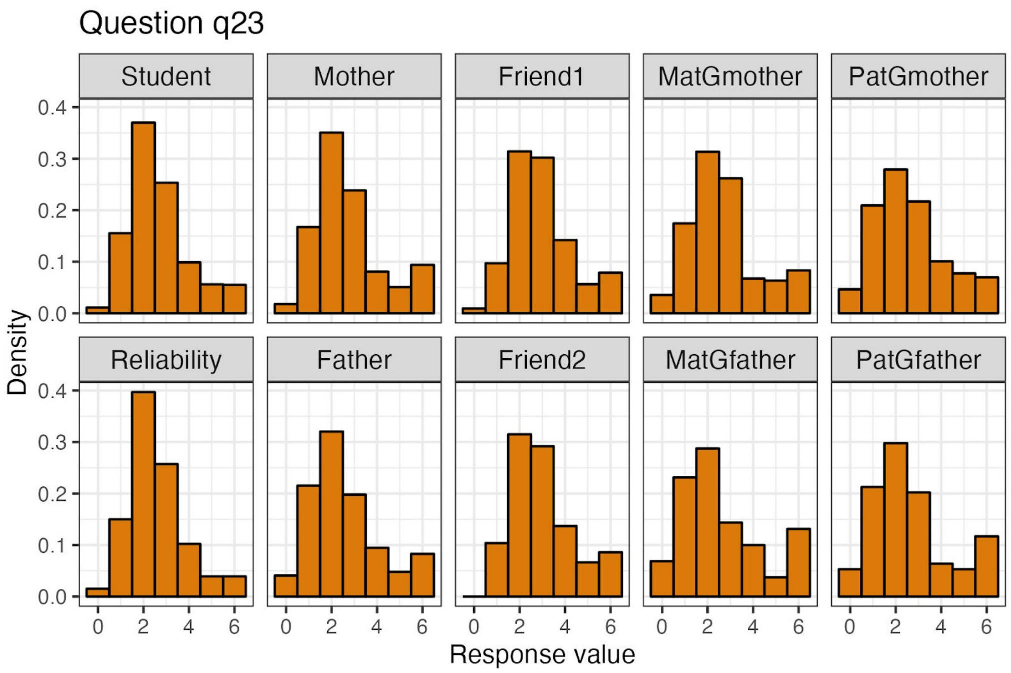

Question q23
Student
Mother
Friend1
MatGmother
PatGmother
Reliability
Father
Friend2
MatGfather
PatGfather
Density
0.0
0.1
0.2
0.3
0.4
0 2 4 6
Response value

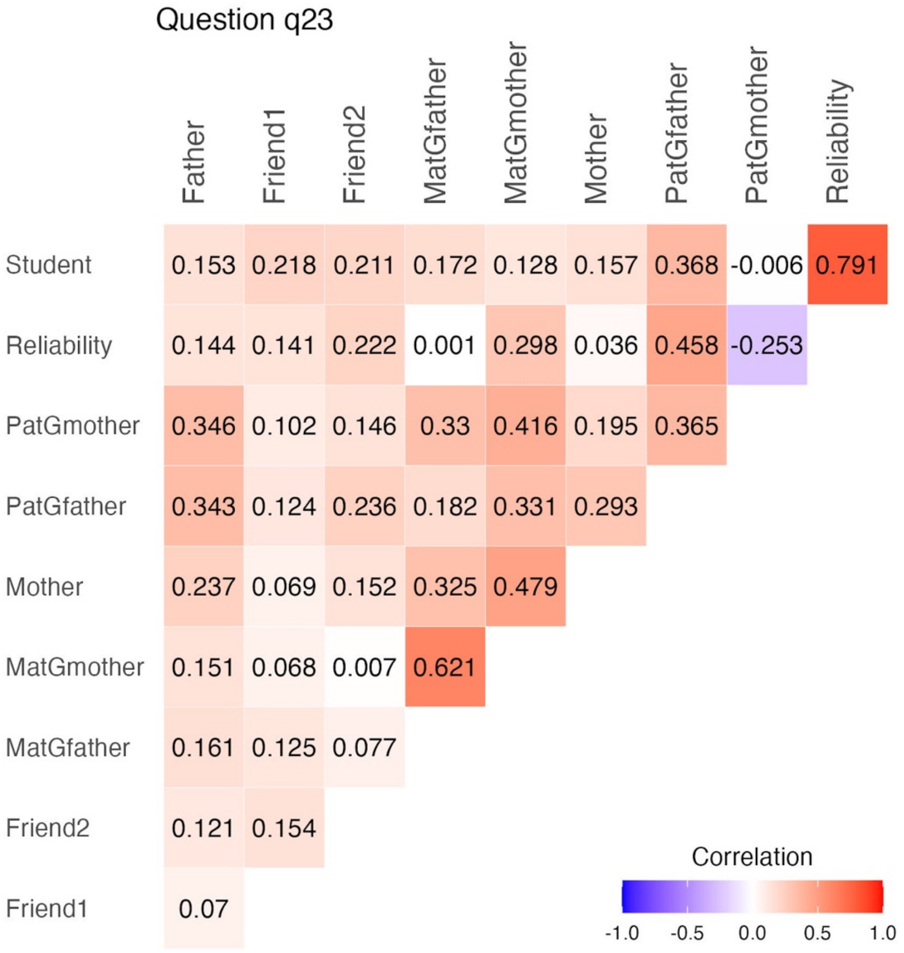

Question q23
Father
Friend1
Friend2
MatGfather
MatGmother
Mother
PatGfather
PatGmother
Reliability
Student 0.153 0.218 0.211 0.172 0.128 0.157 0.368 -0.006 0.791
Reliability 0.144 0.141 0.222 0.001 0.298 0.036 0.458 -0.253
PatGmother 0.346 0.102 0.146 0.33 0.416 0.195 0.365
PatGfather 0.343 0.124 0.236 0.182 0.331 0.293
Mother 0.237 0.069 0.152 0.325 0.479
MatGmother 0.151 0.068 0.007 0.621
MatGfather 0.161 0.125 0.077
Friend2 0.121 0.154
Friend1 0.07
Correlation
-1.0
-0.5
0.0
0.5
1.0

Question 24. How many hours do you spend on social media (e.g., Facebook, Twitter, Instagram)?
Likert 0 to 6; higher values, more hours.

	n	mean	sd	median	min	max	skew	kurtosis	se
Student	1904	3.06	1.19	3	0	6	-0.34	0.35	0.03
Reliability	587	3.06	1.20	3	0	6	-0.60	0.49	0.05
Friend1	1308	3.16	1.21	3	0	6	-0.31	0.23	0.03
Friend2	511	3.11	1.17	3	0	6	-0.43	0.40	0.05
Mother	833	1.92	1.25	2	0	5	0.09	-0.92	0.04
Father	691	1.44	1.29	1	0	6	0.68	-0.06	0.05
MatGfather	160	0.80	1.22	0	0	6	1.55	1.80	0.10
MatGmother	257	1.09	1.33	1	0	6	1.09	0.45	0.08
PatGfather	94	0.55	1.03	0	0	4	1.77	1.81	0.11
PatGmother	130	1.09	1.30	1	0	6	1.08	0.62	0.11

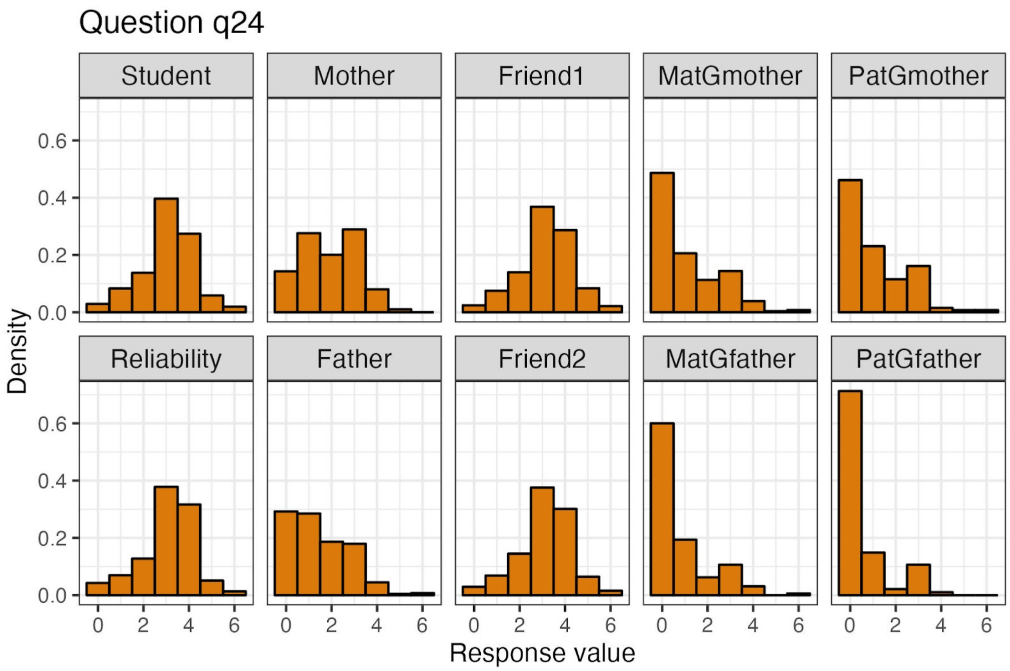

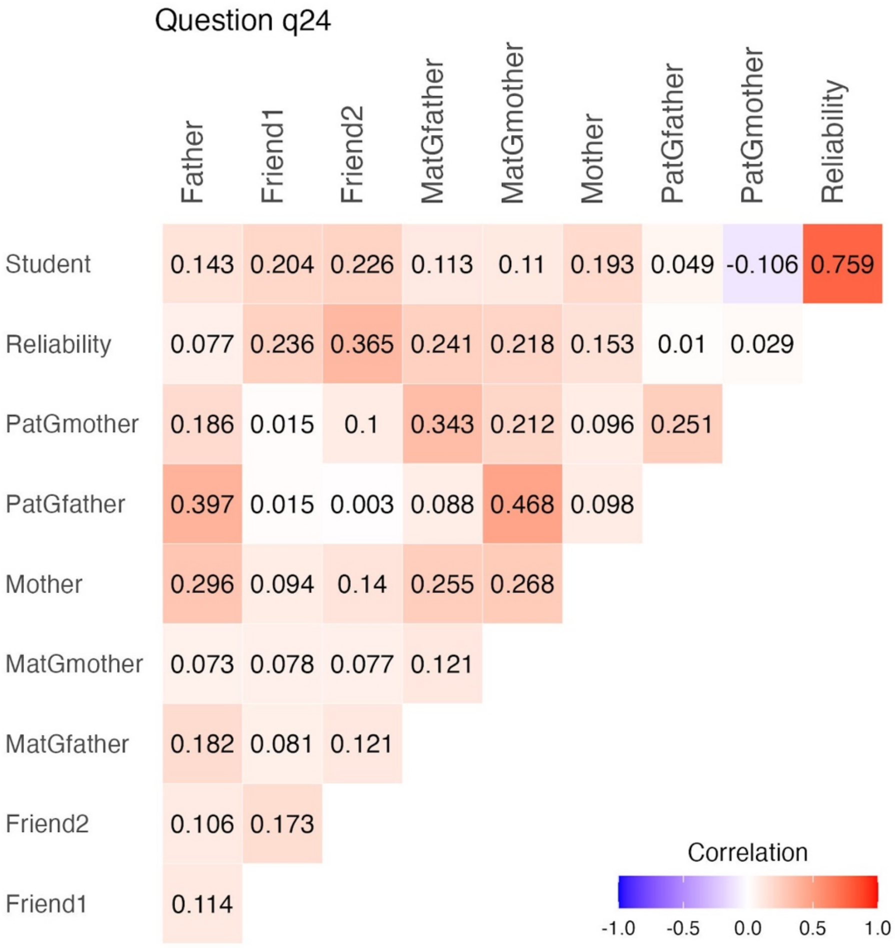

Question 25. How much of your day do you spend interacting with people face-to-face? Likert 0 to 6; higher values, more time.

	n	mean	sd	median	min	max	skew	kurtosis	se
Student	1904	2.09	1.38	2	0	6	0.83	0.45	0.03
Reliability	588	2.03	1.34	2	0	6	0.94	0.76	0.06
Friend1	1309	2.40	1.51	2	0	6	0.60	-0.19	0.04
Friend2	511	2.37	1.47	2	0	6	0.60	-0.11	0.06
Mother	833	2.66	1.67	3	0	6	0.35	-0.73	0.06
Father	691	2.49	1.62	2	0	6	0.40	-0.61	0.06
MatGfather	158	1.85	1.51	2	0	6	1.11	0.94	0.12
MatGmother	256	1.77	1.49	2	0	6	1.12	1.08	0.09
PatGfather	95	1.91	1.55	2	0	6	0.87	0.26	0.16
PatGmother	129	1.89	1.54	2	0	6	0.85	0.21	0.14

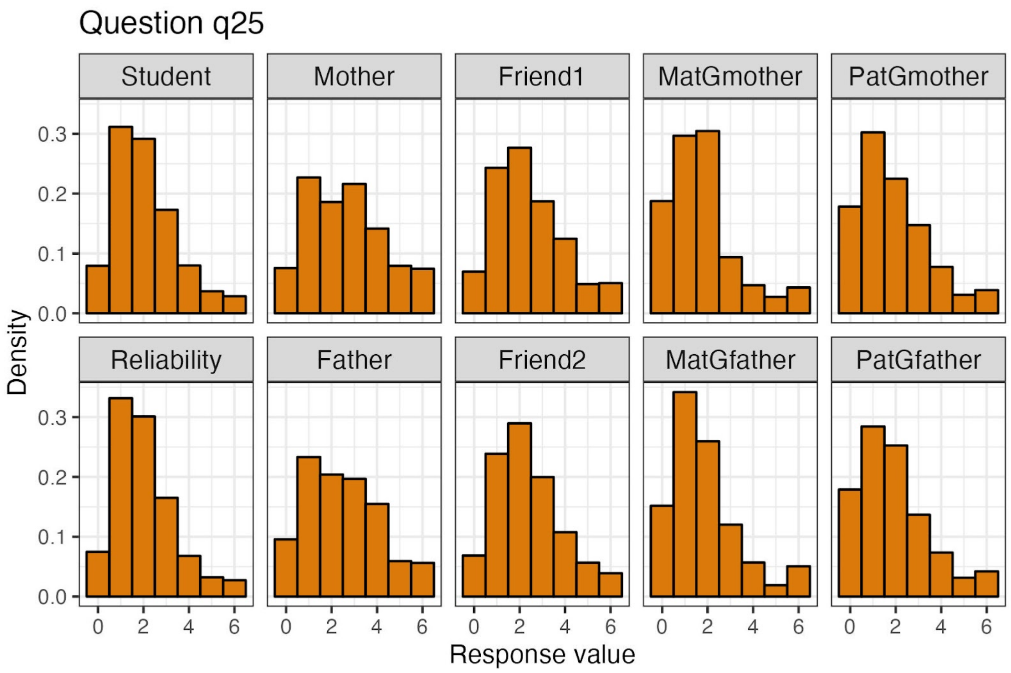
Question q25
Student
Mother
Friend1
MatGmother
PatGmother
Reliability
Father
Friend2
MatGfather
PatGfather
Density
0.0
0.1
0.2
0.3
0
2
4
6
Response value

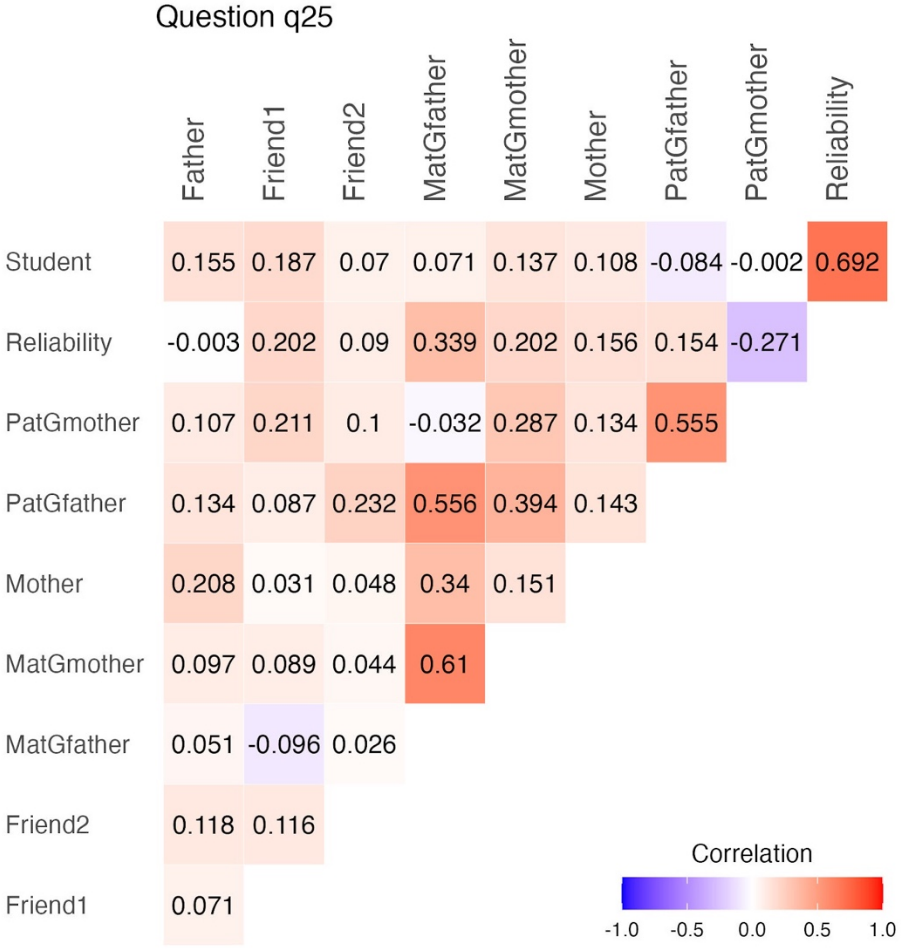
Question q25
Father
Friend1
Friend2
MatGfather
MatGmother
Mother
PatGfather
PatGmother
Reliability
Student 0.155 0.187 0.07 0.071 0.137 0.108 -0.084 -0.002 0.692
Reliability -0.003 0.202 0.09 0.339 0.202 0.156 0.154 -0.271
PatGmother 0.107 0.211 0.1 -0.032 0.287 0.134 0.555
PatGfather 0.134 0.087 0.232 0.556 0.394 0.143
Mother 0.208 0.031 0.048 0.34 0.151
MatGmother 0.097 0.089 0.044 0.61
MatGfather 0.051 -0.096 0.026
Friend2 0.118 0.116
Friend1 0.071
Correlation
-1.0
-0.5
0.0
0.5
1.0

Question 26. Are you concerned about the environment? Likert 0 to 6; higher values, more concerned.

	n	mean	sd	median	min	max	skew	kurtosis	se
Student	1805	4.47	1.23	5	0	6	-0.69	0.41	0.03
Reliability	561	4.50	1.24	5	0	6	-0.75	0.53	0.05
Friend1	1248	4.41	1.34	4	0	6	-0.81	0.62	0.04
Friend2	466	4.39	1.39	4	0	6	-0.79	0.27	0.06
Mother	803	4.50	1.30	5	0	6	-1.00	1.28	0.05
Father	670	4.19	1.41	4	0	6	-0.85	0.46	0.05
MatGfather	151	3.71	1.69	4	0	6	-0.40	-0.71	0.14
MatGmother	245	4.08	1.65	4	0	6	-0.75	-0.04	0.11
PatGfather	90	3.78	1.74	4	0	6	-0.60	-0.48	0.18
PatGmother	119	3.99	1.51	4	0	6	-0.41	-0.50	0.14

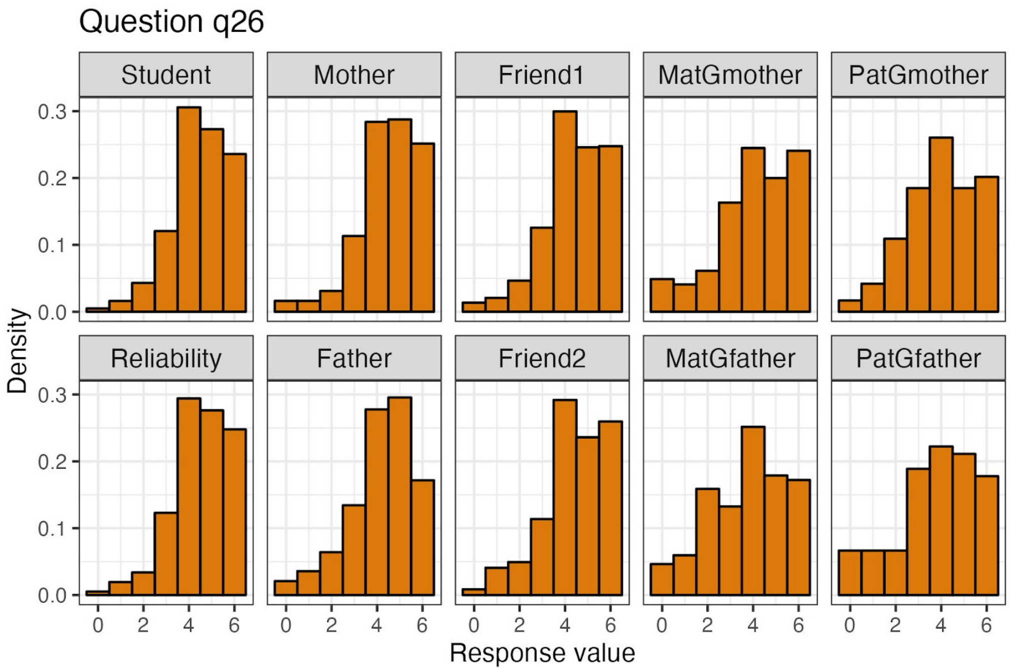

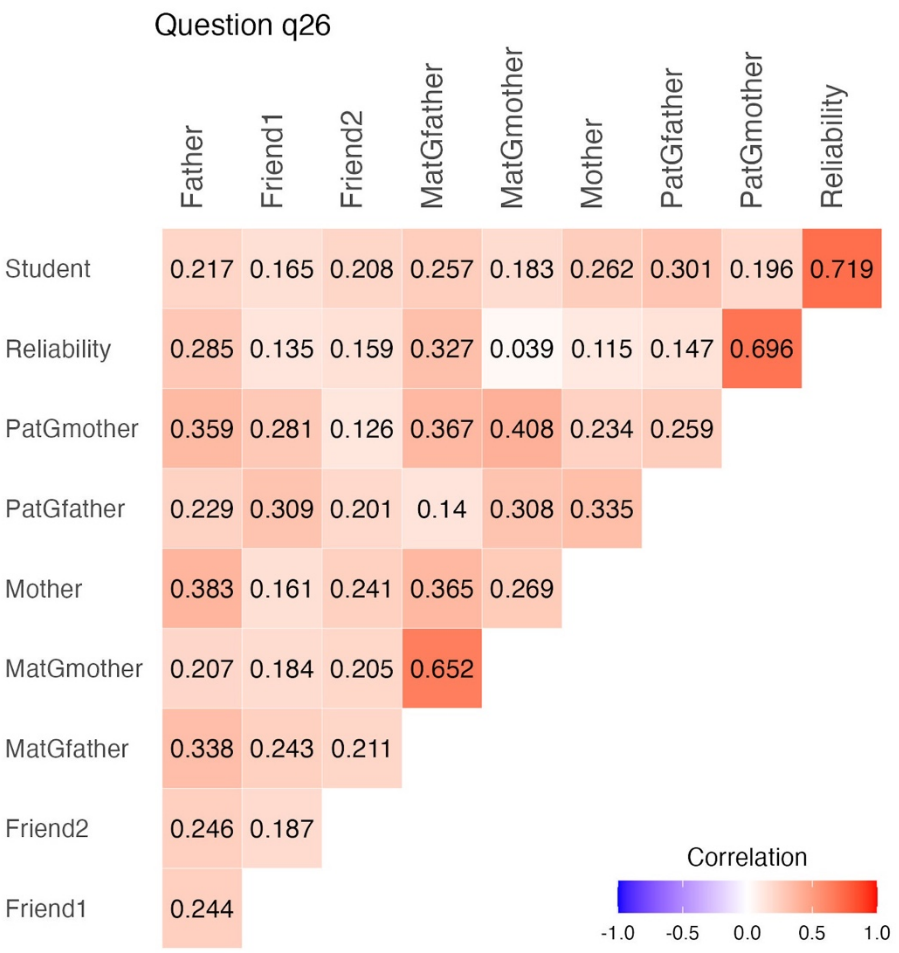

Question 27. Do you take action to help the environment in any of the following ways? Tick all that apply. (Twelve options) Number of activities ticked.

	n	mean	sd	median	min	max	skew	kurtosis	se
Student	1905	5.80	2.43	6.0	0	12	-0.16	-0.22	0.06
Reliability	588	5.96	2.37	6.0	0	12	-0.27	-0.09	0.10
Friend1	1310	5.42	2.56	5.5	0	12	-0.08	-0.41	0.07
Friend2	512	5.29	2.57	5.0	0	12	-0.06	-0.35	0.11
Mother	835	5.58	2.27	6.0	0	12	-0.11	0.08	0.08
Father	696	5.05	2.31	5.0	0	12	-0.17	-0.22	0.09
MatGfather	161	4.69	2.61	5.0	0	12	0.09	-0.34	0.21
MatGmother	260	5.21	2.72	5.5	0	12	-0.20	-0.50	0.17
PatGfather	96	4.42	2.69	4.0	0	12	0.26	-0.34	0.27
PatGmother	130	4.92	2.35	5.0	0	11	-0.05	-0.66	0.21

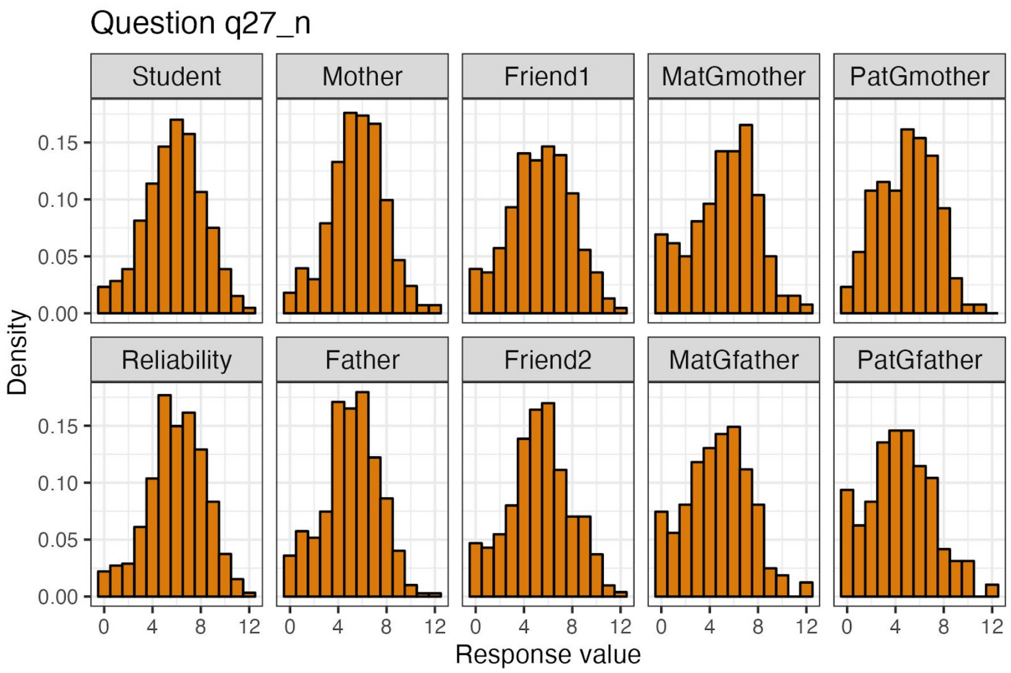
Question q27_n
Student
Mother
Friend1
MatGmother
PatGmother
Reliability
Father
Friend2
MatGfather
PatGfather
Density
0.00
0.05
0.10
0.15
0
4
8
12
Response value

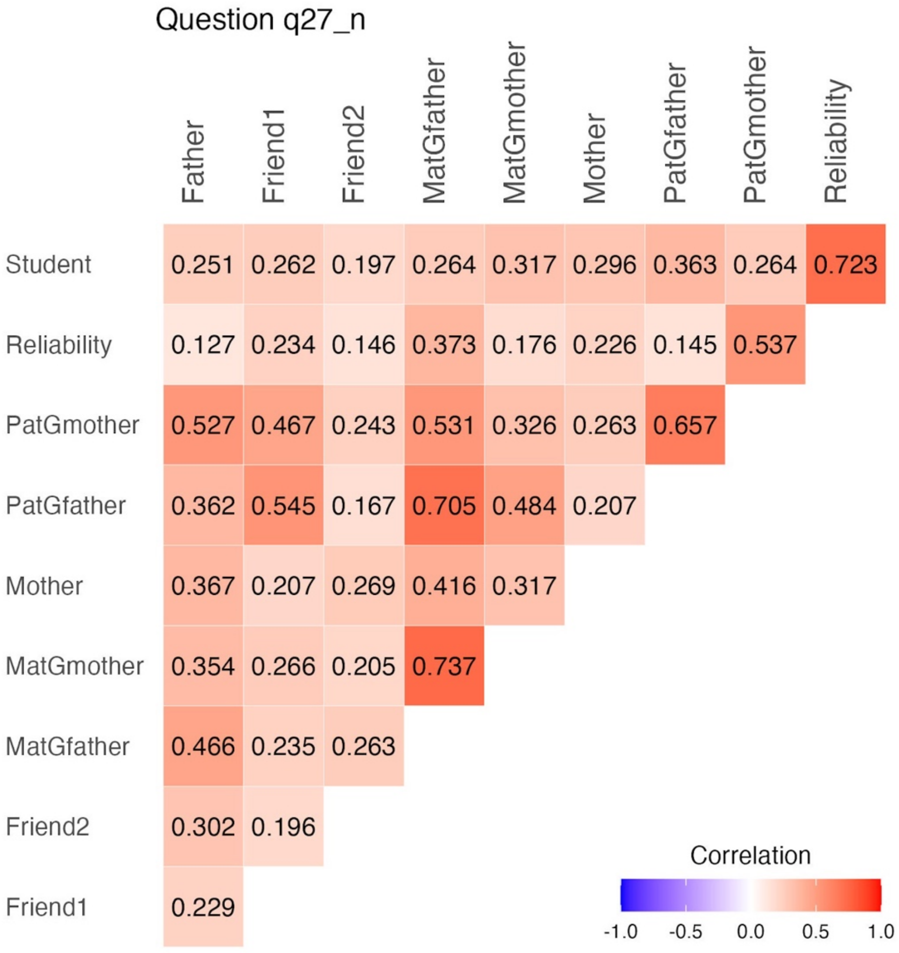
Question q27_n
Father
Friend1
Friend2
MatGfather
MatGmother
Mother
PatGfather
PatGmother
Reliability
Student 0.251 0.262 0.197 0.264 0.317 0.296 0.363 0.264 0.723
Reliability 0.127 0.234 0.146 0.373 0.176 0.226 0.145 0.537
PatGmother 0.527 0.467 0.243 0.531 0.326 0.263 0.657
PatGfather 0.362 0.545 0.167 0.705 0.484 0.207
Mother 0.367 0.207 0.269 0.416 0.317
MatGmother 0.354 0.266 0.205 0.737
MatGfather 0.466 0.235 0.263
Friend2 0.302 0.196
Friend1 0.229
Correlation
-1.0
-0.5
0.0
0.5
1.0
