# Supplementary material for: Cultural transmission of attitudes and behaviours from parents, peers and grandparents
Source: PLoS One. 2026 Jan 28;21(1):e0341433. doi: 10.1371/journal.pone.0341433 (PMC12851453; doi:10.1371/journal.pone.0341433)
Supplement: S6 Table — (PDF) [file pone.0341433.s006.pdf]

S6 Table. Factor loadings

Factor loadings and communalities.

Item	Summary	Factor Loadings								h^2
		Relig.	Politic	Environ.	Health	Music	Read.	Screen	Social	
1	Belief in a god/higher power	0.87	0.03	0.06	0.10	0.05	−0.05	0.01	0.01	0.72
2	Identifies with religion	0.76	0.11	0.02	0.05	0.03	−0.06	−0.01	0.02	0.60
3	Religious attendance	0.72	0.10	0.07	−0.02	−0.01	0.00	0.02	0.08	0.54
4	Political orientation	0.11	0.62	−0.18	−0.01	−0.03	0.04	−0.03	0.12	0.52
5	Orientation of preferred party	0.06	0.72	−0.10	−0.05	0.01	0.04	0.01	0.16	0.54
6	Political activity level	0.02	−0.10	0.32	−0.15	−0.04	0.06	−0.01	0.10	0.17
9	Exercise frequency	−0.07	−0.14	−0.26	0.56	−0.04	0.06	−0.13	0.41	0.46
10	Importance of healthy lifestyle	0.15	−0.05	−0.09	0.69	0.03	0.04	−0.14	0.27	0.54
11	Number of exercises practiced	−0.06	0.00	0.04	0.19	0.01	0.02	0.07	0.45	0.27
12	Average sleep	−0.08	0.01	0.02	0.09	0.00	−0.01	0.06	−0.04	0.02
13	Smoking level	−0.04	−0.01	−0.03	−0.22	−0.02	0.05	−0.06	0.01	0.05
14	Alcohol use	−0.27	0.08	0.06	−0.06	0.04	−0.08	−0.08	0.16	0.11
15	Meatiness of diet	−0.02	0.13	−0.24	0.00	0.04	0.01	0.07	−0.04	0.11

16	Fruit/veg intake	−0.04	0.04	0.19	0.28	−0.04	0.06	−0.09	0.10	0.20
17	Video media watch time	−0.01	0.03	−0.05	−0.08	−0.16	0.10	0.77	0.16	0.49
18	Video media enjoyment	0.05	−0.01	0.04	0.14	0.06	−0.03	0.61	0.00	0.41
19	Reading time	−0.04	0.09	−0.05	−0.16	−0.08	1.05	0.09	0.07	1.00
20	Reading enjoyment	−0.01	0.02	0.18	0.07	0.14	0.58	0.01	−0.15	0.48
21	Music listening time	−0.04	−0.03	−0.07	−0.19	0.47	0.08	0.07	0.32	0.51
22	Music enjoyment	0.06	0.00	−0.01	0.12	1.09	0.01	−0.17	−0.08	1.00
23	Number of close friends	0.00	0.07	0.04	0.03	−0.01	0.01	0.09	0.30	0.09
24	Social media time	0.02	−0.21	−0.05	−0.11	0.06	−0.09	0.37	0.34	0.43
25	Face-to-face interaction time	0.03	0.03	0.06	0.01	−0.01	−0.03	−0.02	0.20	0.05
26	Environmentalism concern	0.05	−0.10	0.57	0.15	0.09	0.01	0.04	0.01	0.47
27	Number of pro-environ. habits	−0.01	0.09	0.75	0.04	−0.01	0.00	0.06	0.14	0.59

Note. Salient loadings (> .5) have darker shading and marginal ones (> .2), lighter shading. Green loadings are positive and red are negative.
