# Supplementary material for: Cultural transmission of attitudes and behaviours from parents, peers and grandparents
Source: PLoS One. 2026 Jan 28;21(1):e0341433. doi: 10.1371/journal.pone.0341433 (PMC12851453; doi:10.1371/journal.pone.0341433)
Supplement: S7 Text — (PDF) [file pone.0341433.s007.pdf]

S7 Text. Descriptives of text-based answers

Table A Descriptives question 2. What is your religion? (8 options + “other”, leading to a text box). Only items of frequency >= 20 listed.

Religion	Freq	Religion	Freq
Atheist	1472	Hindu	136
Agnostic	1226	Uniting	107
Catholic	1147	Jewish	57
Christian	728	Spiritual	39
Anglican	387	Orthodox	31
Buddhist	259	Sikh	26
Islam	178	No religion	21

Table B Descriptives question 5. What is your preferred Australian political party? Name one. Only items of frequency >= 10 listed.

Pol. Party	Freq	Pol. Party	Freq
Labor	1741	Democrat	19
Coalition	1689	One Nation	17
Greens	1115	Conservative	13
Christian	46	Independent	12

Table C Descriptives question 7. Are you a fan of a particular spectator sport? (6 options + “other”, leading to a text box). Only items of frequency >= 10 listed.

Sport	Freq	Sport	Freq	Sport	Freq
None	1920	Badminton	56	Golf	20
AFL	1316	Motor	55	Gymnastics	20
Soccer	630	Hockey	53	Ice-skating	19
Tennis	480	Volleyball	40	Athletics	18
Basketball	409	Swimming	34	Surf	17
Cricket	287	e-sport	30	Equestrian	11
Rugby	140	Martial	25	NFL	11
Netball	75	Cycling	20	Table-tennis	10

Table D Descriptives question 8. Are you a fan of a particular sports team or player? (Yes or No). If yes, please specify. Only items of frequency >= 15 listed.

Team or player	Freq	Team or player	Freq	Team or player	Freq
No	3348	Arsenal	34	Geelong	23

Eagles	584	Carlton	31	LA Lakers	20
Dockers	328	Chelsea	31	L. James	19
Liverpool	77	India cricket	29	Essendon	18
Man. United	76	Collingwood	26	Scorchers	18
R. Federer	72	W Coast Fever	25	All Blacks	17
Aus. cricket	38	Hawthorn	24	Glory	16
Wildcats	35	R. Nadal	24	N Melbourne	16

Table E Descriptives question 15. How would you describe your diet? Tick all that apply (6 options + “other”, leading to a text box). Only items of frequency >= 5 listed.

Pol. Party	Freq	Pol. Party	Freq
None	4884	Halal	39
Vegetarian	341	Flexitarian	26
Pescatarian	213	Keto	23
Health_restrictions	165	NRM	17
Vegan	140	Protein	13
Healthy	42	Meat	8

Table F Descriptives question 11. What are your main forms of exercise? Tick all that apply. (15 options + “other”, leading to a text box). Only items of frequency >= 20 listed.

Sport	Freq	Sport	Freq	Sport	Freq
Walking	4920	Tennis	328	Rugby	70
Gym	2926	Martial arts	285	Pilates	63
Running	2243	Golf	264	Frisbee	39
Cycling	1111	AFL	193	Gardening	38
Swimming	1110	Yoga	169	Skating	28
Dancing	784	Cricket	120	Rowing	26
Netball	462	Badminton	80	Surfing	24
Basketball	403	Hockey	78	Climbing	23
Soccer	379	Volleyball	75	Boxing	20

Table G Descriptives question 27. Do you take action to help the environment in any of the following way? Tick all that apply. (12 options). All items listed.

Action to help the environment	Freq
Use reusable shopping bags	5453
Recycle plastics/glassware/cans/etc	5172
Switch off household lights/appliances when not in use	5109

Limit water/electricity use	3767
Eat locally produced food	3490
Use public transport instead of driving	2519
Ride or walk instead of driving	2499
Limit meat consumption	2278
Select offset carbon footprint when given the option	1876
Compost vegetable scraps	1858
Donate to environmental groups/causes	1227
Volunteer time to conservation groups/tree planting…	454
