# Supplementary material for: Cultural transmission of attitudes and behaviours from parents, peers and grandparents
Source: PLoS One. 2026 Jan 28;21(1):e0341433. doi: 10.1371/journal.pone.0341433 (PMC12851453; doi:10.1371/journal.pone.0341433)
Supplement: S8 Text — (PDF) [file pone.0341433.s008.pdf]

## S8 Text. Resemblance by question

All correlation and resemblance values by agent-pair and by factor can be round in Fig. A.

Resemblance values (z-scores of the correlations calculated with a Monte Carlo method) for each question are in Figure A. Seven of the questions in the survey were text-based and had to be numerically recoded. Five of those (Q2, Q5, Q7, Q8, Q15) involved selection of one choice or entering text, while two (Q11 and 27) allowed respondents to select multiple choices; Q11 accepted, in addition to choices, typed input.

To obtain Resemblance in one-choice text questions for each pair of social agents, we counted the number of times the two agents' answers matched (veridical count). Then we obtained 10,000 scrambled counts by randomizing the order of the answers from one of the agents and counting the number of matches. We then calculated the z-score of the veridical count in the distribution of scrambled counts. For multiple-choice questions, Resemblance was calculated in the same way, but counting, for each pair of individuals, the number of single item matches in their two answers.

Resemblance values tend to be higher for text-based questions than the corresponding numeric questions. Text is more nuanced, and reveals more similarity within groups. For example, the forms of exercise selected by participants in Q11_text and the environment-helping activities selected in Q27_text, are much more heritable than the number of forms of exercise (Q11_numeric) and the number of activities (Q27_numeric). Similarly, participants are much more similar within networks when looking at the religion participants selected: religion (Q2_text) is more heritable than the dichotomised religiosity recoded in Q2_numeric; selected political party (Q5_text) is more heritable than ordinally recoded Q5_numeric; and selected diet type (Q15_text) than the ordinally recoded Q15_numeric. Questions Q7 and Q8 had not been numerically recoded.

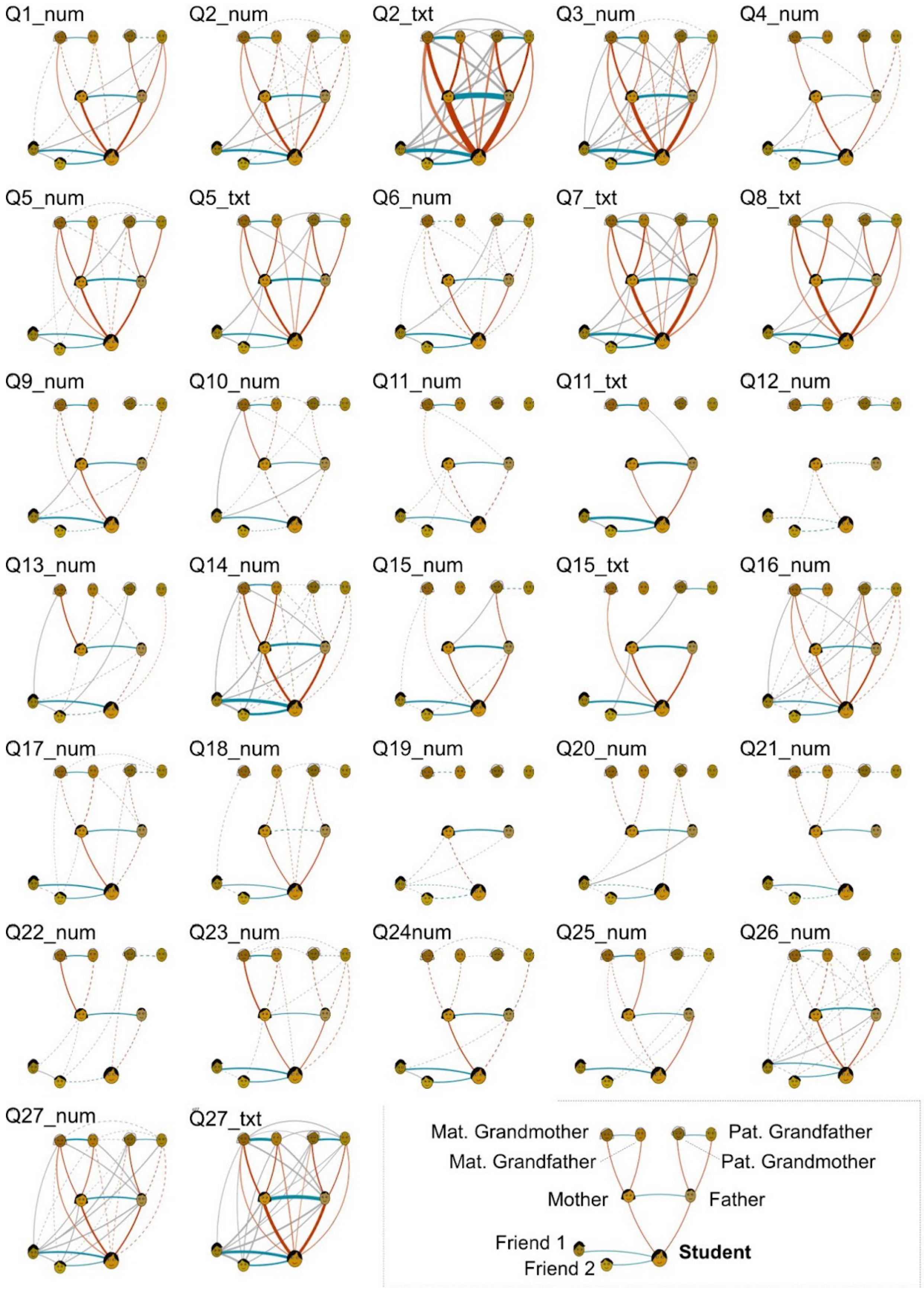


Figure A. Resemblance values for each of the numeric or numerically recoded questions for all pairs of social agents (bottom left panel identifies agents). Line

thickness is proportional to Resemblance. Red lines: direct vertical transmission. Orange lines: indirect vertical transmission. Blue lines: horizontal transmission. Grey lines: no obvious transmission pathway. Multiple comparison correction applied; only z-scores with corrected $p < 0.05$ are plotted. Dashed lines: $p<0.05$; solid lines: $p < 0.001$.
