# Supplementary material for: Cultural transmission of attitudes and behaviours from parents, peers and grandparents
Source: PLoS One. 2026 Jan 28;21(1):e0341433. doi: 10.1371/journal.pone.0341433 (PMC12851453; doi:10.1371/journal.pone.0341433)
Supplement: S9 Text — (PDF) [file pone.0341433.s009.pdf]

S9 Text. Parental Type Analysis and Maximum Likelihood Estimation of Vertical, Horizontal and Other biases

We estimated the additive similarity of parents and friends on one hand, and students' dichotomised traits on the other.

Table A. P and B values for all parental types and all factors.

Factor	Parental Type*	P = Number of networks with this parental type	N = Number of students with the trait with this parental type	P−N = Number of students with the opposite trait with this parental type	Transmission coefficient (trait) B = N / P	Transmission coefficient (opposite trait) B = (P−N) / P
environment	0	185	71	114	0.384	0.616
environment	1	119	61	58	0.513	0.487
environment	2	122	66	56	0.541	0.459
environment	3	156	111	45	0.712	0.288
health	0	134	37	97	0.276	0.724
health	1	206	97	109	0.471	0.529
health	2	81	44	37	0.543	0.457
health	3	161	108	53	0.671	0.329
music	0	101	76	25	0.752	0.248
music	1	39	33	6	0.846	0.154
music	2	282	210	72	0.745	0.255
music	3	160	130	30	0.812	0.188
politics	0	131	14	117	0.107	0.893
politics	1	264	85	179	0.322	0.678
politics	2	48	8	40	0.167	0.833
politics	3	139	70	69	0.504	0.496
reading	0	206	66	140	0.32	0.68
reading	1	172	69	103	0.401	0.599
reading	2	75	24	51	0.32	0.68
reading	3	129	58	71	0.45	0.55
religion	0	164	14	150	0.085	0.915
religion	1	172	79	93	0.459	0.541
religion	2	61	14	47	0.23	0.77

religion	3	185	136	49	0.735	0.265
screen	0	164	83	81	0.506	0.494
screen	1	56	34	22	0.607	0.393
screen	2	245	164	81	0.669	0.331
screen	3	117	88	29	0.752	0.248
social	0	141	65	76	0.461	0.539
social	1	58	37	21	0.638	0.362
social	2	243	156	87	0.642	0.358
social	3	140	108	32	0.771	0.229

*Parental type codes:

	Trait	Opposite Trait
0	HV	hv
1	Hv	hV
2	hV	Hv
3	hv	HV

We estimated the biases that explain the similarity between our student trait-state (have the trait or not have the trait) and their parents', their friends' as well as others' trait-states, for each question or factor. A MLE simulation obtained the combinations of three parameters: vertical bias, horizontal bias and other bias, capturing the contagion from or to parents, friends and anyone else (e.g., other people, school, church, social media and other social institutions, individual learning, etc), for each factor. MOVE the rest of this paragraph to DISCUSSION? Any differences between questions or factors indicate (a) that social contagion is different across areas of behaviour and attitudes and (b) what these differences are for the topics covered in our survey.

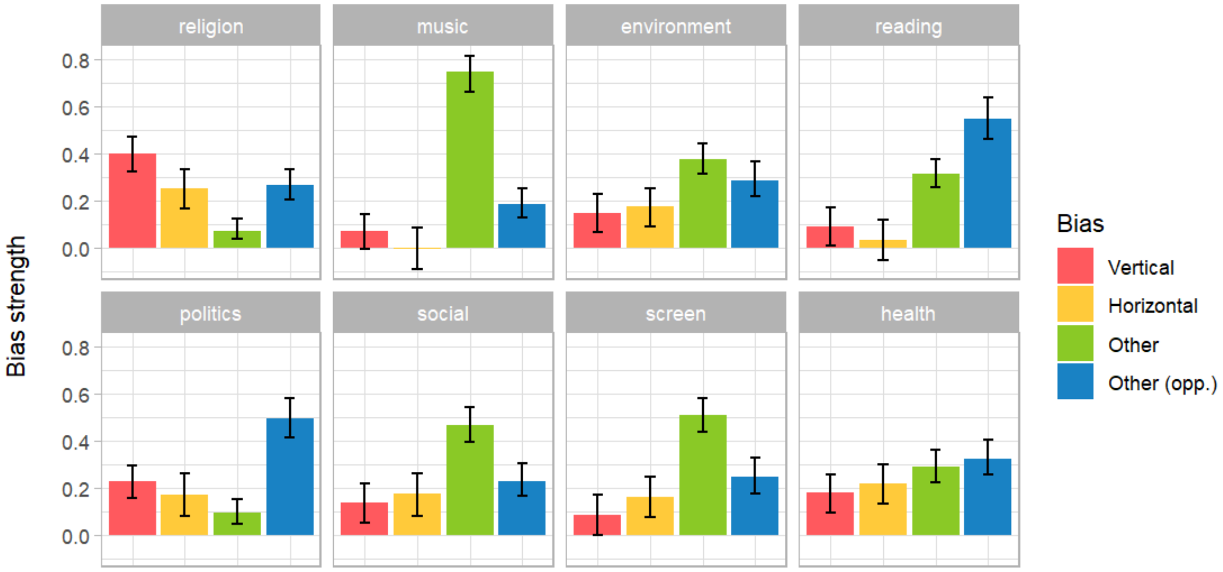

Figure A. Alternative visualisation of the same data in Figure 6: Vertical, Horizontal and Other bias values for the trait and the opposite trait related to each factor, here as a bar plot with 95% CIs.
