# Supplementary material for: Cultural transmission of attitudes and behaviours from parents, peers and grandparents
Source: PLoS One. 2026 Jan 28;21(1):e0341433. doi: 10.1371/journal.pone.0341433 (PMC12851453; doi:10.1371/journal.pone.0341433)
Supplement: S10 Text — (PDF) [file pone.0341433.s010.pdf]

S10 Text. Prevalence of traits in students by parental type by question

In order to study the vertical (parents) and horizontal (friends) influences on the answers to each question, we subsetted, for each question, the networks for which at least the student, one parent and one friend had answered that question. We then recoded these answers to establish whether the student, their parents and their friends had the trait assessed in the question (e.g. believe in God) or not. A student was assigned trait = 1 if their answer was above the median answer to that question, and trait = 0 if their answer was below the median. If the answer was equal to the median, 1 was assigned when the distribution had positive skewness, and 0 when the distribution had negative skewness. To code parents and friends, the same criteria were applied to the averaged answers from both parents (or from one parent, if only one answered the survey), or both friends (or from one friend, if only one answered the survey). Finally, we categorised networks into one of four cultural parental types:

1. Neither parents nor friends have the trait
2. Only parents have the trait
3. Only friends have the trait
4. Both parents and friends have the trait

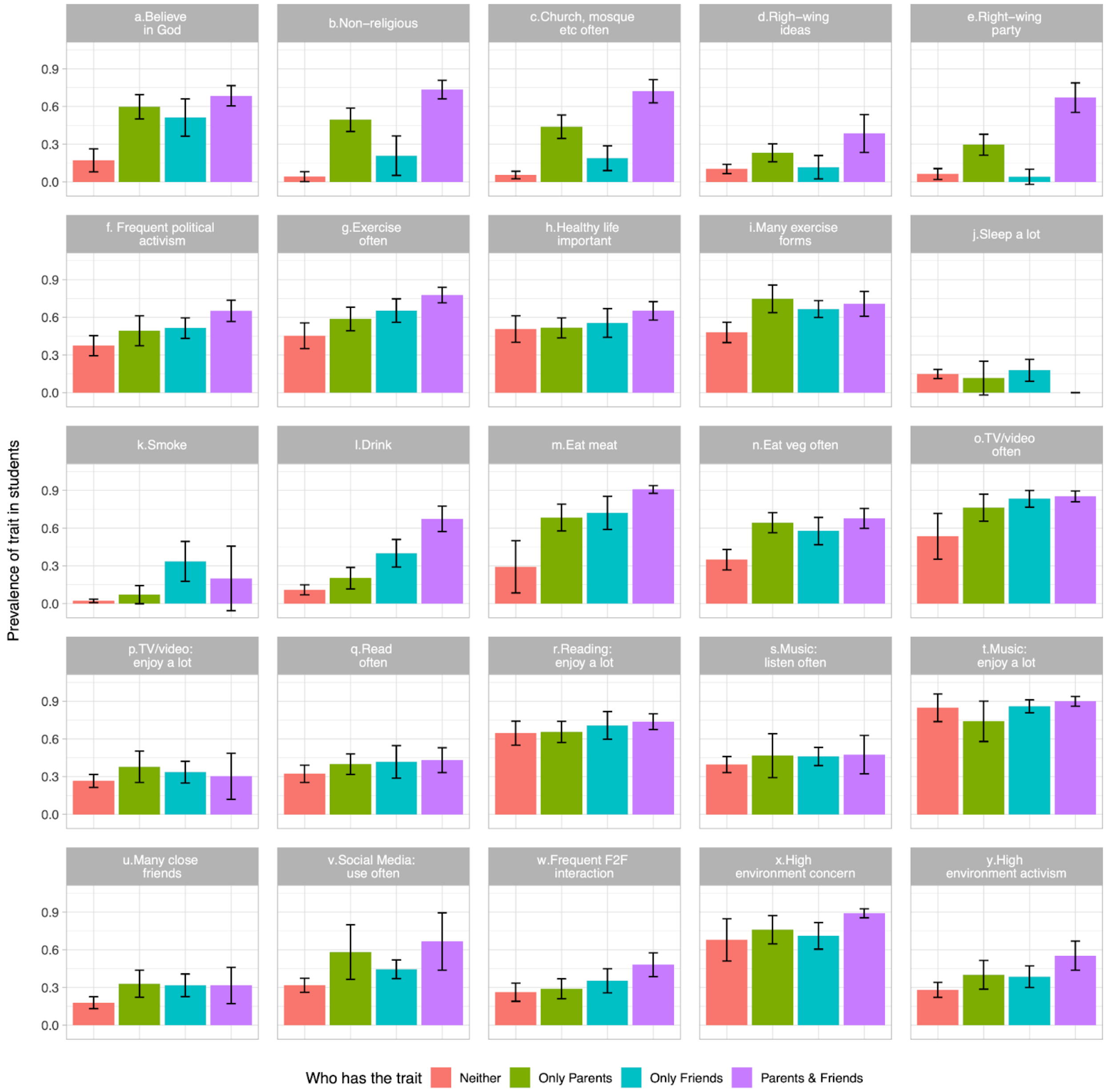

Figure A. Proportion of students who had the trait assessed in each question in our survey, by cultural parental type. E.g., if the question asked "do you believe in God", this figure gives the proportions of students whose answers were above the median and therefore had a strong belief in God (mean and bootstrapped 95% CI, N=10,000).

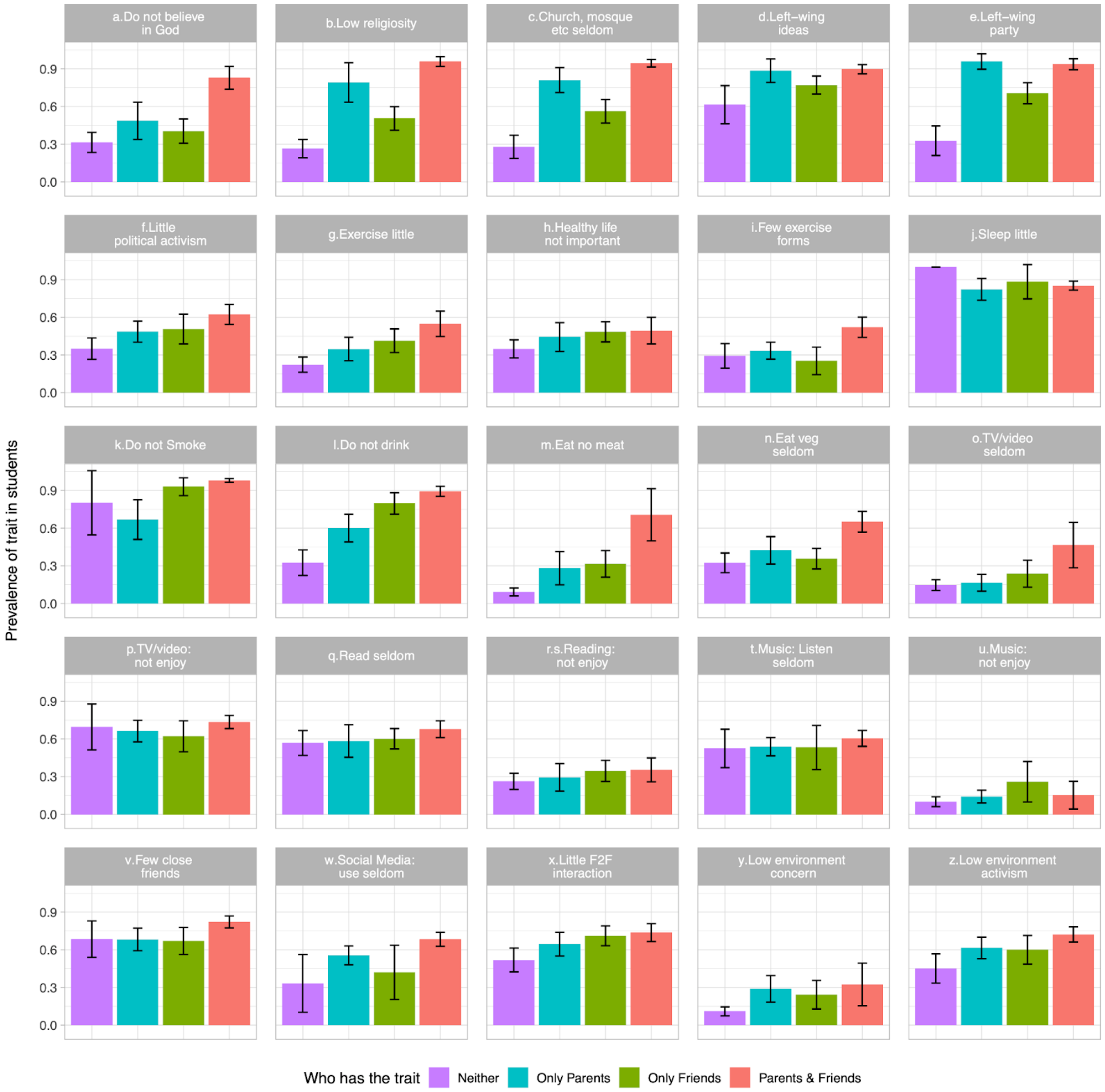

Figure B. Proportion of students who did not have the trait assessed in each question in our survey, by cultural parental type. E.g., if the question asked “do you believe in God”, this figure gives the proportions of parents and students whose answers were below the median and therefore had a weak belief in God (mean and bootstrapped 95% CI, N=10,000).
